# cuTauLeaping: A GPU-Powered Tau-Leaping Stochastic Simulator for Massive Parallel Analyses of Biological Systems

**DOI:** 10.1371/journal.pone.0091963

**Published:** 2014-03-24

**Authors:** Marco S. Nobile, Paolo Cazzaniga, Daniela Besozzi, Dario Pescini, Giancarlo Mauri

**Affiliations:** 1 Dipartimento di Informatica, Sistemistica e Comunicazione, Università degli Studi di Milano-Bicocca, Milano, Italy; 2 Dipartimento di Scienze Umane e Sociali, Università degli Studi di Bergamo, Bergamo, Italy; 3 Dipartimento di Informatica, Università degli Studi di Milano, Milano, Italy; 4 Dipartimento di Statistica e Metodi Quantitativi, Università degli Studi di Milano-Bicocca, Milano, Italy; 5 Istituto di Analisi dei Sistemi ed Informatica “Antonio Ruberti”, Consiglio Nazionale delle Ricerche, Roma, Italy; 6 SYSBIO Centre for Systems Biology, Milano, Italy; Virginia Tech, United States of America

## Abstract

Tau-leaping is a stochastic simulation algorithm that efficiently reconstructs the temporal evolution of biological systems, modeled according to the stochastic formulation of chemical kinetics. The analysis of dynamical properties of these systems in physiological and perturbed conditions usually requires the execution of a large number of simulations, leading to high computational costs. Since each simulation can be executed independently from the others, a massive parallelization of tau-leaping can bring to relevant reductions of the overall running time. The emerging field of General Purpose Graphic Processing Units (GPGPU) provides power-efficient high-performance computing at a relatively low cost. In this work we introduce cuTauLeaping, a stochastic simulator of biological systems that makes use of GPGPU computing to execute multiple parallel tau-leaping simulations, by fully exploiting the Nvidia's Fermi GPU architecture. We show how a considerable computational speedup is achieved on GPU by partitioning the execution of tau-leaping into multiple separated phases, and we describe how to avoid some implementation pitfalls related to the scarcity of memory resources on the GPU streaming multiprocessors. Our results show that cuTauLeaping largely outperforms the CPU-based tau-leaping implementation when the number of parallel simulations increases, with a break-even directly depending on the size of the biological system and on the complexity of its emergent dynamics. In particular, cuTauLeaping is exploited to investigate the probability distribution of bistable states in the Schlögl model, and to carry out a bidimensional parameter sweep analysis to study the oscillatory regimes in the Ras/cAMP/PKA pathway in *S. cerevisiae*.

## Introduction

Nowadays, the use of computational methods represents a valuable and integrative tool to conventional experimental biology, thanks to the promising capability to gain a global-level understanding of the emergent dynamics of biological systems and to elucidate the mechanisms governing their functionality, that most of the times can be hardly determined by laboratory experiments only. Indeed, the computational study of biological systems can present many advantages in terms of cost, ease to use and rapidity, and can support laboratory work by suggesting *ad hoc* designed experiments. In this context, mathematical modeling tools, simulation algorithms and analysis techniques simplify the predictions on the way these complex systems behave in normal conditions and how they react to genetic, chemical or environmental perturbations; moreover, they can facilitate the verification of specific dynamical properties, which can be characterized by non linear or multistable phenomena [1]–[4].

Given a mathematical model describing the interactions between the components of a biological system, computer algorithms allow to validate and analyze the model, giving the possibility to recreate *in silico* a wide spectrum of emergent phenomena; in particular, simulation algorithms are an essential tool to study the temporal evolution of biological systems. Anyway, the shift from the reproduction of the experimental observations to the capability of making predictions on the behavior of the system in unexplored conditions can be limited by the lack or the inaccuracy of available quantitative data (e.g., reaction rates, intracellular concentrations, etc.), which are indispensable to settle a good model parameterization. To cope with these problems, several computational methods can be exploited [1], such as parameter estimation (PE) [5]–[9], sensitivity analysis (SA) [10]–[12], parameter identifiability (PI) [13]–[15], parameter sweep analysis (PSA) [6], reverse engineering (RE) [7], [16], etc. These methods usually require the execution of many simulations to explore the high-dimensional search space of possible model parameterizations, therefore resulting in prohibitive computational costs.

An additional aspect that should be considered when defining mathematical models of biological systems is related to the experimental evidences that most of the cellular regulation networks, especially those involving few amounts of some molecular species, are affected by noise [17]. The randomness occurring at the molecular scale can induce stochastic phenomena at the macromolecular scale, giving rise to non deterministic behaviors. The classical modeling approach based on ordinary differential equations (ODEs) is not able to fully capture all the effects of stochastic processes; in this context, the most remarkable example is the phenomenon of bistability, that can be effectively investigated by means of stochastic approaches [10], [18].

Stochastic modeling of biological systems can rely on the definition of stochastic differential equations (SDEs), like the Langevin equation [19], or on the stochastic formulation of chemical kinetics [20], whereby a biological system is formalized by specifying the set of molecular species which interact through a set of chemical reactions based on mass-action kinetics. These reaction-based models can be simulated by means of Monte Carlo procedures, like Gillespie's stochastic simulation algorithm (SSA) [20], which was proven to be equivalent to the Chemical Master Equation and to generate an exact temporal evolution of well-stirred biochemical systems [21]. Since SSA proceeds by simulating the execution of a single reaction per computation step, it may require a high running time even for small systems. Many improvements to the original SSA procedure were proposed [22]–[24], but all of them still result computationally expensive; among stochastic simulation algorithms, one of the most efficient is tau-leaping [25], which outperforms SSA by allowing the execution of multiple reactions per step, thus providing a relevant reduction of the running time. In the last years, tau-leaping was extended in order to avoid the possibility of generating negative molecular amounts [26], [27], to tackle the problem of stiffness [28], or to keep into account the spatial localization of molecular species [29] and delayed reactions [30]. The present work is based on the modified tau-leaping version proposed in [27].

Despite the computational improvements brought by the tau-leaping algorithm, a typical task for the analysis of stochastic models can still be affected by high computational costs: as a matter of fact, besides requiring many different simulations to explore the space of all possible model parameterizations with PE, SA, PI or PSA analysis, the application of stochastic simulation algorithms needs a congruous number of repetitions of the simulations, under the same conditions, in order to deal with the effects of noise due to stochastic fluctuations and to obtain statistically significant results about the system behavior. Therefore, for the analysis of biological systems based on stochastic modeling approaches, an efficient strategy for the parallelization of multiple tau-leaping executions is necessary to obtain a consistent reduction of the computational costs, and to provide scientists with a powerful tool that may speed up the achievement of new insights into the functioning of biological systems.

The traditional methods to perform parallel executions of an algorithm consist in multithreading [31], distributed computing on clusters [32], [33], or custom circuitry produced with Field Programmable Gate Arrays (FPGA) [34]. The emerging area of General Purpose Graphics Processing Units (GPGPU) computing is a halfway solution that gives access to the huge unexpressed computational power of modern video cards, which reside in almost any personal computer of mid-range price. In addition, GPGPU computing is not only beneficial from a computational point of view, but it also allows to strongly reduce the energy consumption. As a matter of fact, in June 2013, two GPU-powered machines (EURORA and Aurora Tigon) reached the first places in the Green500 list of the most power-efficient supercomputers (http://www.green500.org/lists/green201306). Both machines are based on NVIDIA Tesla K20 GPU.

Previous works presented the parallelization on Graphics Processing Units (GPU) of multiple SSA simulations [35]–[37], also considering reaction-diffusion systems [38], and the fine-grain acceleration of a single tau-leaping execution [39]. The research we describe here represents the first achievement, to the best of our knowledge, in running a huge number of parallel tau-leaping simulations on the GPU for the analysis of biological systems; to this aim, we introduce the novel stochastic simulator called cuTauLeaping. We discuss, in particular, some relevant issues related to the optimization of the tau-leaping implementation on GPU, since a simple and naïve porting to the CUDA architecture of its working process turned out to be inefficient. In cuTauLeaping we introduce a novel restructuring of tau-leaping workflow – consisting in the execution of four different algorithmic phases – which better fits the GPU architecture and avoids the inefficiency drawbacks. We also present the design of *ad hoc* data structures that are necessary for an appropriate memory allocation on the GPU: this is a particularly tricky issue in GPGPU computing, since GPU memories need to be properly used to achieve good computational performances. Another drawback of GPU computing is related to the choice of efficient, parallel and statistically sound random number generators (RNGs). Like any other Monte Carlo-based algorithm, tau-leaping heavily relies on high-quality pseudorandom sequences. In this work, we discuss how to obtain good computational performances by exploiting a proper RNG among the available and out-of-the-box solutions.

In order to compare the computational costs of cuTauLeaping with respect to a standard CPU-based implementation of the original tau-leaping algorithm, we carry out different batches of simulations of four stochastic models of real biological systems: the Michaelis-Menten kinetics [40], a prokaryotic gene regulatory network [35], [41], the Schlögl model [42], and the Ras/cAMP/PKA signal transduction pathway in yeast [6], [43], [44]. In particular, we exploit cuTauLeaping to execute massive parallel simulations to investigate the probability distribution of bistable states in the Schlögl model, and to perform a fine-grain bidimensional parameter sweep analysis for the identification of oscillatory regimes in the Ras/cAMP/PKA pathway in *S. cerevisiae*. In addition, we exploit synthetic systems of increasing size, randomly generated as described in [39], to evaluate the impact of the models size on the computational performance, irrespective of any actual dynamical properties (i.e., oscillations, bistability, etc.) that the systems may present.

We show that even with a limited number of parallel simulations (ranging from 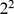 to 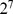, according to the complexity of the investigated biological system), cuTauLeaping outperforms the CPU implementation of tau-leaping with an empirical speedup ranging from 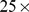 up to 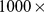, approximately, therefore portending its valuable application for thorough analyses of stochastic biological systems.

## Methods

### Stochastic modeling and simulation of chemical kinetics

According to the stochastic formulation of chemical kinetics [20], a model of a biological system 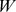 is defined by specifying the set of 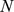 molecular species 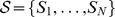 which interact through 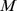 chemical reactions 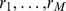; 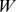 is assumed to be spatially homogeneous and in thermal equilibrium within a fixed volume 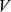. A generic reaction 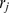 is defined as
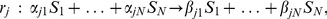
(1)where 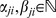 are the stoichiometric coefficients associated, respectively, to the 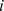-th reactant and to the 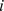-th product of the 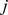-th reaction, for 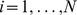 and 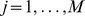. Reactions 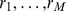 implicitly define two matrices, 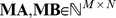, having 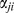 and 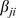 as elements, respectively. We denote by 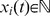 the number of molecules of species 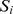 present in 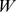 at time 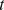, so that 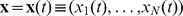 represents the state of the system at time 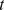. We denote by 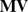 the state change matrix associated to 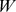, defined as 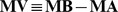. Each row of this matrix, 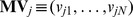, is called the state change vector, and consists of elements 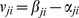, 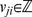, that represent the stoichiometric change of species 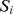 due to reaction 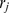.

In addition to 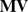, we define a supplementary state change matrix 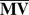, where 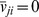 for each 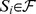, for a given 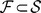 that contains the molecular species whose amounts have to be kept constant during the simulation; the subset 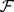 is used to account for a continuous “feed” of molecules into the system. This condition can be used to mimic, for instance, the non-limiting availability of some chemical resources, or the execution of *in vitro* buffering experiments, in which an adequate supply of some species is introduced in 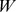 in order to keep their quantity constant [45].

The traditional way to calculate the stochastic temporal evolution of 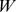 consists in solving the so-called Chemical Master Equation (CME), which describes the probability distribution function associated to 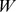 [46]. Numerical solution algorithms for the CME are usually based on matrix descriptions of the discrete-state Markov process [47]; anyway, these methods are computationally expensive and not always feasible, especially for systems consisting of many molecular species, for which the number of reachable states is huge or even (countably) infinite. Several analytical solution algorithms for the CME exist, for instance those based on uniformization methods [48]–[50], finite state projection algorithms [51], [52] or the sliding window method [53]; other methods were also introduced for special reaction systems characterized by particular initial conditions (see, e.g., [54] and references therein). A different strategy to solve the CME consists in generating trajectories of the underlying Markov process. A method of this type is the stochastic simulation algorithm (SSA) [20], [55], which provides exact realizations of the associated continuous time, discrete state space jump Markov process 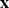 of a biochemical system 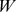, whose initially conditioned density function is determined by the CME itself; as such, SSA is logically equivalent to the CME [21].

Briefly, starting from the system state 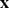, SSA determines which reaction will be executed during the next time interval 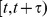, by calculating the probability of each reaction 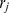 to occur in the next infinitesimal time step 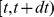. This probability is proportional to 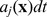, being 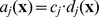 the *propensity function* of reaction 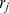, where 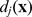 is the number of distinct combinations of the reactant molecules occurring in 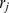 and 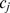 is a stochastic constant encompassing the physical and chemical properties of 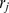 [55]. The time 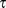 before a reaction takes place is chosen according to the following equation:
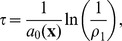
where 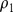 is a random value sampled in [0,1] with a uniform probability, and 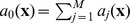. The index 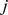 of the reaction to be executed is the smallest integer in 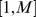 such that
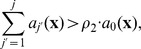
where 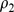 is a random value sampled in [0,1] with a uniform probability.

In [25] an approximate but faster version of SSA, called tau-leaping, was introduced for the purpose of reducing the computational burden typical of SSA. SSA and tau-leaping share the characteristic that, even starting from the same initial state of the system, repeated executions of the algorithms will produce (usually quantitative, but potentially also qualitative) different temporal dynamics, thus reflecting the inherent noise of the system. These two algorithms, anyway, differ with respect to the way reactions are applied at each step: in SSA, only *one* reaction is applied, while with tau-leaping *several* reactions can be applied.

### Tau-leaping algorithm

We present here the main features of tau-leaping, that are beneficial to illustrate the choices at the basis of the GPU implementation proposed in this work. We refer to [25], [56] for further details and, especially, to [27], which describes the improved version of the tau-leaping algorithm considered here.

Given a state 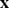 of the system 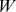, let 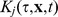 denote the exact number of times that a reaction 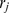 would be fired in the time interval 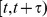; 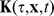 denotes the probability distribution vector having 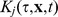 as elements. For arbitrary values of 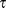, the computation of the values 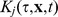 can be as difficult as solving the corresponding CME. On the contrary, if 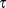 is small enough so that the change in the state 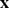 during 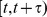 is so slight that no propensity function will suffer an appreciable change in its value (this is called the *leap condition*), then it is possible to evaluate a good approximation of 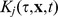 by using the Poisson random variables with mean and variance 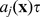. So doing, the stochastic temporal evolution of the system is no longer exact (as in the case of SSA); however, the accuracy of tau-leaping can be fixed a priori by means of an error control parameter 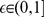, which is involved in the computation of the changes in the propensity functions and of the time increment 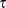. The propensity functions change as a consequence of the modification in the molecular amounts of the reactant species, therefore the leap condition must be verified after each state update. This is achieved by evaluating an additional quantity 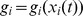 for each species 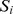, which is related to the highest order 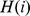 of the reactions in which 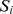 is involved as a reactant (see [27] for details). This information, along with the number of molecules of 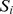 involved in all highest-order reactions (given by the system state 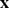), is then used to bound the relative change of 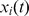. Starting from the state 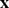 and choosing a 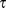 value that satisfies the leap condition, the state of the system at time 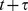 is updated according to
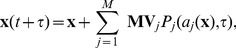
(2)where 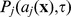 denotes an independent sample of the Poisson random variable with mean and variance equal to 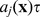.

Note that the execution of many reactions per step could lead to negative amounts of the molecular species in 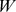 [25]. To be more precise, if the reactions executed during a step consume a number of reactant molecules greater than those occurring in the system, then negative species amounts would be generated; therefore, the simulation step cannot be executed. To avoid these situations, some reactions are considered as *critical*: a reaction 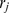 is marked as critical if there are not sufficient reactant molecules to fire it at least 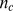 times in the next time interval. In this work we use the threshold 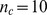, as suggested in [27]. At each iteration of tau-leaping, all reactions are partitioned into the sets of non-critical reactions (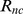) and critical reactions (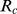). Only a single reaction belonging to 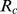 – selected following the SSA procedure – is allowed to fire during 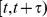.

The length of the step 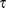 satisfying the leap condition is calculated as
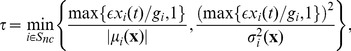
(3)where 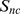 is the set of indices of reactant species not involved in critical reactions, and the values 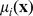 and 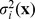 are computed as follows:
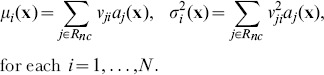
(4)


If the execution of a tau-leaping step would lead to negative amounts of some species, then the 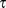 value is halved and the number of reactions to execute is sampled *ex novo*.

Finally, if 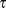 is smaller than a multiple of 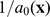 – which corresponds to the average time increment of SSA – then a certain number of SSA steps is executed because, given the actual state of the system, this will be more accurate and efficient than a tau-leaping step.

### General-purpose GPU computing

The emerging field of GPGPU computing allows developers to exploit the great computational power of modern multi-core GPUs, by giving access to the underlying parallel architecture that was conceived for speeding up real-time three-dimensional computer graphics. The GPU implementation of tau-leaping that we propose in this work was developed and optimized for Nvidia's Compute Unified Device Architecture (CUDA), a cross-platform GPGPU library that combines the Single Instruction Multiple Data (SIMD) architecture and multi-threading. CUDA automatically handles the control flow divergence, that is, threads can take different execution paths in a transparent way for the programmer. Nevertheless, conditional branches should be avoided whenever possible as they cause a reduction of performances, due to the serialization of the execution until reconvergence. For this reason, the tau-leaping algorithm required a major reconstruction in order to reduce the need for conditional branches, as will be described in the Results section.

### CUDA

**Figure 1:**
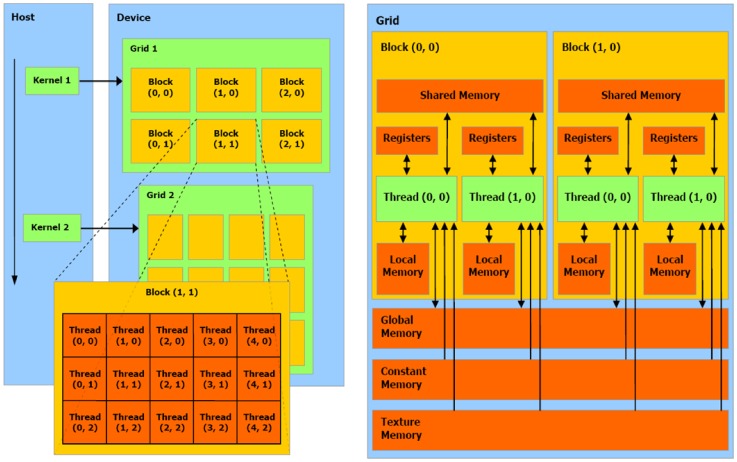
Schematization of CUDA architecture. Schematic representation of CUDA threads and memory hierarchy. *Left side*. Thread organization: a single kernel is launched from the host (the CPU) and is executed in multiple threads on the device (the GPU); threads can be organized in three-dimensional structures named blocks which can be, in turn, organized in three-dimensional grids. The dimensions of blocks and grids are explicitly defined by the programmer. *Right side*. Memory hierarchy: threads can access data from many different memories with different scopes; registers and local memories are private for each thread. Shared memory let threads belonging to the same block communicate, and has low access latency. All threads can access the global memory, which suffers of high latencies but is cached since the introduction of Fermi architecture. Texture and constant memories can be read from any thread and feature a cache as well. Figures are taken from the Nvidia's CUDA programming guide [58].

Using CUDA's naming conventions, the programmer implements a kernel (that is, a C function) loaded from the host (the CPU) to the devices (one or more GPUs), replicated in many copies named threads. Threads can be organized in three-dimensional structures named blocks which, in turn, are contained in three-dimensional grids. Whenever the host runs a kernel, the GPU creates the corresponding grid and automatically schedules each block on one streaming multiprocessor (SM) available on the GPU, a solution that allows a transparent scaling of performances on different devices (see Figure 1, left side). CUDA poses limitations on the number of threads a block may contain: up to 1024 threads can be distributed in the three dimensions, and each dimension must not exceed 512 threads. The SM organizes scheduled blocks in batches consisting in 32 parallel threads, called warps. Since more than one block can be assigned at once to the same SM, a warp scheduler manages the execution of warps.

Threads can read and write data from different kinds of memories (Figure 1, right side): the global memory (visible from all threads), the shared memory (accessible from threads belonging to the same block), and the local memory (registers and arrays, accessible from the owner thread). Furthermore, all threads can read data from two cached memories: the constant memory and the texture memory. CUDA offers other types of memory, like the page-locked memory, portable memory, and mapped memory; as our implementation does not exploit these additional features, they go beyond the scope of the present paper and will not be further described here.

The global memory is generally very large (up to thousands MBs), but suffers from high access latencies, whilst the shared memory is faster but much smaller (tens of thousands KBs for each SM). Being a very small resource on each multiprocessor, the shared memory poses constraints on the blocks size, thus limiting the number of simultaneous threads that can be executed at once. However, in order to achieve the best performances, the shared memory represents a precious resource that must be exploited as much as possible. These considerations are central in our implementation of tau-leaping and will be described in more detail in the Results section.

Since the introduction of the Fermi architecture, the global memory features a small amount of cache, which makes the use of the texture memory counterproductive [57]. This cache resides on the same on-chip memory (64 KB for each SM) that is used for both cache and shared memories, and gives the programmer the opportunity to balance the two memory amounts. Our GPU implementation of tau-leaping was optimized for the Fermi architecture, since it heavily relies on the availability of shared memory for performance reasons.

### Random numbers generation

Stochastic simulation algorithms exploit random numbers generators (RNGs). Nvidia's software development kit [58] contains several libraries and utilities that help developers in the process of creating software for this architecture; CURAND is a RNG library which allows the GPU-based generation of random deviates that can be used both by the host (via memory copy) or directly by the device. CURAND is the only external library that we exploited in cuTauLeaping, while the remaining code was developed in plain vanilla CUDA code.

CURAND from CUDA toolkit v5.0 [59] gives access to many different RNGs that can produce pseudo-random sequences with a very large period: XORWOW [60], MRG32K3A [61] and Mersenne Twister (MT) [62]. Among these RNGs, MT is the one that yields the longest pseudo-random sequences thanks to its 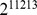 period, while XORWOW and MRG32K3A generate sequences of pseudo-random numbers with a period of 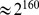 and 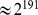, respectively. MT was not used for the implementation of cuTauLeaping because it has three drawbacks: 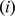 at most 256 threads per block can operate simultaneously [59], 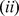 the memory footprint is larger than the other generators [63], 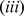 it is much slower than the other two algorithms.

XORWOW is faster than MRG32K3A, but it is known to present statistical flaws [64] and it is rejected by 3 of the 106 tests of the BigCrush statistical test suite [65]. CURAND exploits these RNGs to generate random deviates with uniform or standard normal distributions; since the introduction of the CUDA toolkit v5.0, CURAND libraries offer the possibility to generate the Poisson-distributed random deviates required by tau-leaping. cuTauLeaping offers the possibility to perform the simulations using both RNGs; the results presented in what follows are based on MRG32K3A.

## Results

In this section we describe the development of cuTauLeaping and its application to perform parallel stochastic simulations in a massively parallel way, by running multiple independent simulations as parallel CUDA threads. We introduce our GPU-oriented design of tau-leaping, consisting in a four phases workflow, and present the data structures, the memory allocation strategies and the advanced functions exploited on the Fermi architecture.

We compare the computational performances of cuTauLeaping with the CPU implementation of tau-leaping provided in the software COPASI, a well known application for the simulation and the analysis of biochemical networks [66]. To this aim, we exploit as benchmarks four stochastic models of biological systems of increasing complexity, formally described in Text S1. In addition, to analyze the influence of the size of the model (i.e., number of reactions and molecular species) on the performances of cuTauLeaping, we executed several tests on randomly generated synthetic models characterized by different size and various parameterizations.

Finally, we show the advantages of using cuTauLeaping to investigate the effects of systematic perturbations on the system dynamics. To this aim, we performed different parameter sweep analyses (PSA) on the Schlögl and Ras/cAMP/PKA models, in which one (PSA-1D), two (PSA-2D) or three (PSA-3D) parameters were simultaneously varied within given sweep intervals (chosen with respect to a fixed reference value for each parameter). Within these ranges, the numerical values of each varied parameter were determined with a linear sampling for the amounts of molecular species; a logarithmic sampling was instead considered for stochastic constants (if not stated otherwise), in order to uniformly span over many orders of magnitude. All PSA were performed by generating a set of different initial conditions – corresponding to different parameterizations of the model under investigation – and then automatically executing the parallel stochastic simulations with cuTauLeaping.

### Design and implementation of cuTauLeaping

In cuTauLeaping, the workflow of the traditional tau-leaping algorithm is partitioned in different phases, which altogether allow a better exploitation of the parallel architecture of the GPU than a monolithic implementation. The rationale behind this choice is that the resources on each SM are limited, thus they would be quickly consumed by the data structures employed by tau-leaping, causing a low occupancy of the GPU that would then result in worse performances. Moreover, since tau-leaping embeds the potential execution of SSA simulation steps, a “fat” kernel responsible for both simulation algorithms would not be convenient, because of the following issues: 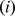 when the largest permissible time step 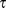 for non-critical reactions is very low, it is faster to forgo tau-leaping and to execute an arbitrary number of SSA steps (see Step 3 in the modified Poisson tau-leaping algorithm presented in [27]); 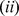 SSA is simpler and requires fewer resources than tau-leaping (the only thing the two algorithms share is the vector of propensity functions). Therefore, the partitioning of tau-leaping workflow in different phases allows a faster execution of the simulations, thanks to the reduced memory footprint, which yields a higher level of parallelism.

**Figure 2:**
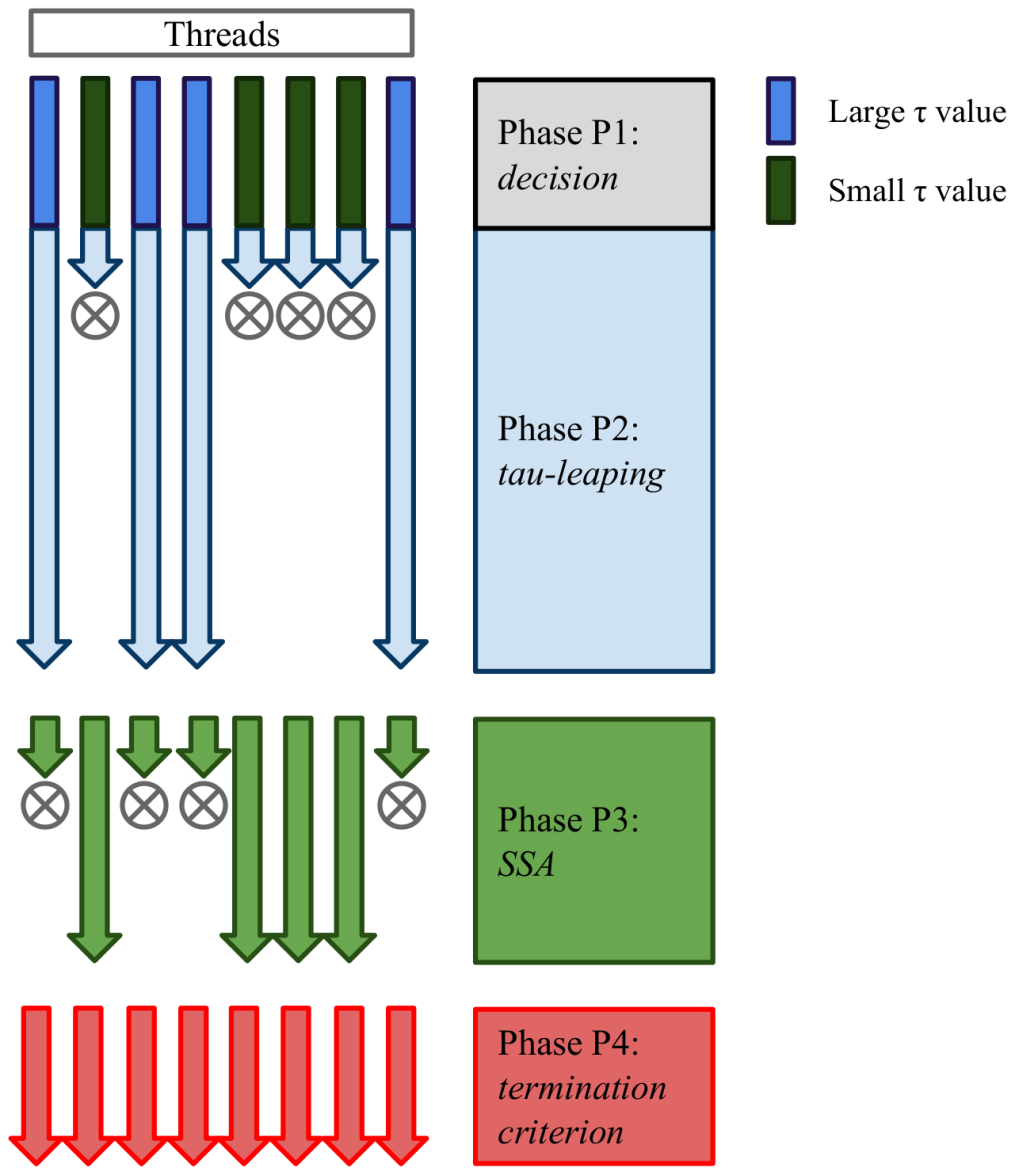
cuTauLeaping workflow. Simplified scheme of cuTauLeaping workflow: in phase P1 each thread calculates the value 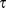 for the simulation step; in phase P2, the threads whose 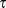 is “large” perform a tau-leaping step **(by executing a set of non-critical reactions and (possibly) one critical reaction)**; the remaining threads perform a fixed number of SSA steps **(where one reaction is executed at each step)** during phase P3. The phases are iterated until all threads have reached 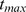, a termination criterion verified during phase P4.

In this section we describe in detail the design and the implementation of the different CUDA kernels that stand at the basis of cuTauLeaping. The four phases that constitute cuTauLeaping, schematized in Figure 2, are:

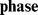
 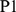: each thread 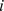, where 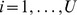, and 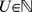 is specified by the user, determines a tentative value for the length of the time step 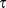 for the non-critical reactions, by using Equation (3);

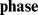
 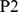: all threads where the length of the time step is such that 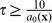 execute a tau-leaping step;

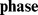
 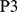: the remaining threads execute a fixed number of SSA steps (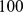 in our default setting);

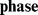
 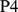: check the termination criterion of the simulation in all threads (cuTauLeaping termination).

Each thread proceeds by applying tau-leaping or SSA steps, which are mutually exclusive, according to the value of a vector 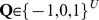, where for each 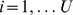 the element 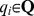 is set to 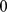 if the 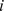-th thread must execute SSA, 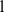 if the 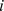-th thread must execute tau-leaping, while the value 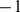 corresponds to the signal of terminated simulation.

In cuTauLeaping the first two phases are implemented in a single kernel, so that the tau-leaping step can be executed right after the calculation of the 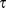 value, without the need for a global memory write (e.g., to update 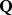) or a recalculation of the propensity functions and 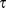 (that could be required, in such a case), which would reduce the performances. In particular, during the first two phases, after the computation of the putative 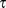 value for non-critical reactions, a second putative time step value related to critical reactions is calculated, and the smallest one is used in the current tau-leaping step. If the first putative 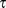 value is used, then only non-critical reactions are sampled from the Poisson distributions and applied; otherwise, besides non-critical reactions, also one critical reaction is selected and applied (as described in [27]).

The four phases are implemented in the following kernels, which are executed in a sequential manner by each thread 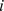:

- kernel 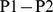: if 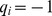, then terminate the kernel; otherwise, calculate the 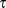 value for non-critical reactions.

If 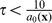, then 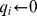 and terminate the kernel; else 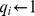 and execute a tau-leaping step updating the system state 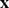 (according to Equation 2, by executing a set of non-critical reactions and, possibly, one critical reaction) and the global simulation time (by setting 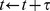). If 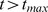, then 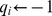 and terminate the kernel;

- kernel 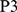: if 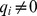, then terminate the kernel; otherwise, perform the SSA steps (by executing a single reaction at each step), and update the system state 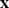 (according to Equation 2) and the global simulation time 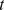 (by setting, at each SSA step, 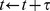). If 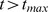 set 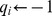 and terminate the kernel;
- kernel 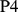: if 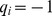 (for all threads), then terminate cuTauLeaping; else go back to kernel 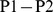.

**Figure 3:**
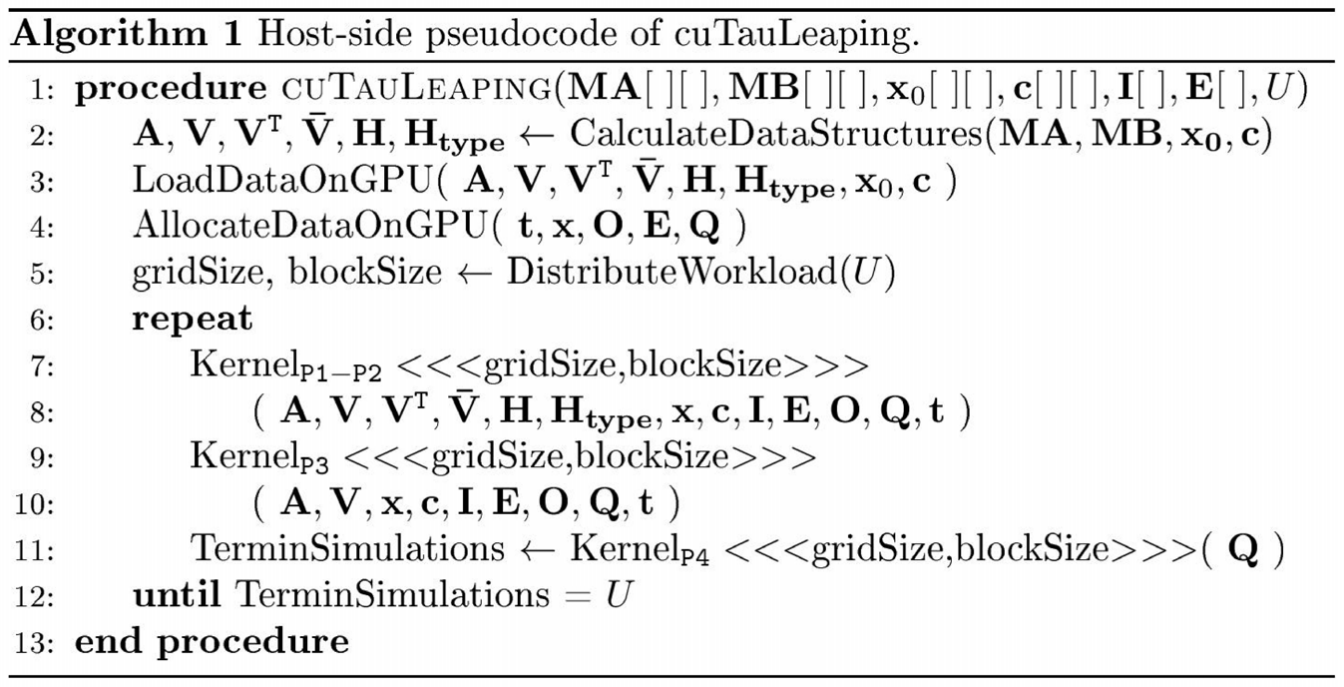
Pseudocode of cuTauLeaping – host side. Host-side pseudocode of cuTauLeaping. As a first step, the stoichiometric information of the reactions is exploited to pre-calculate the data structures needed by the algorithm; all matrices are flattened during this process. Then, once the support memory areas are allocated (e.g., the chunk of global memory where the system dynamics will be stored), the four phases of cuTauLeaping begin and are repeated until all simulations are completed.

**Figure 4:**
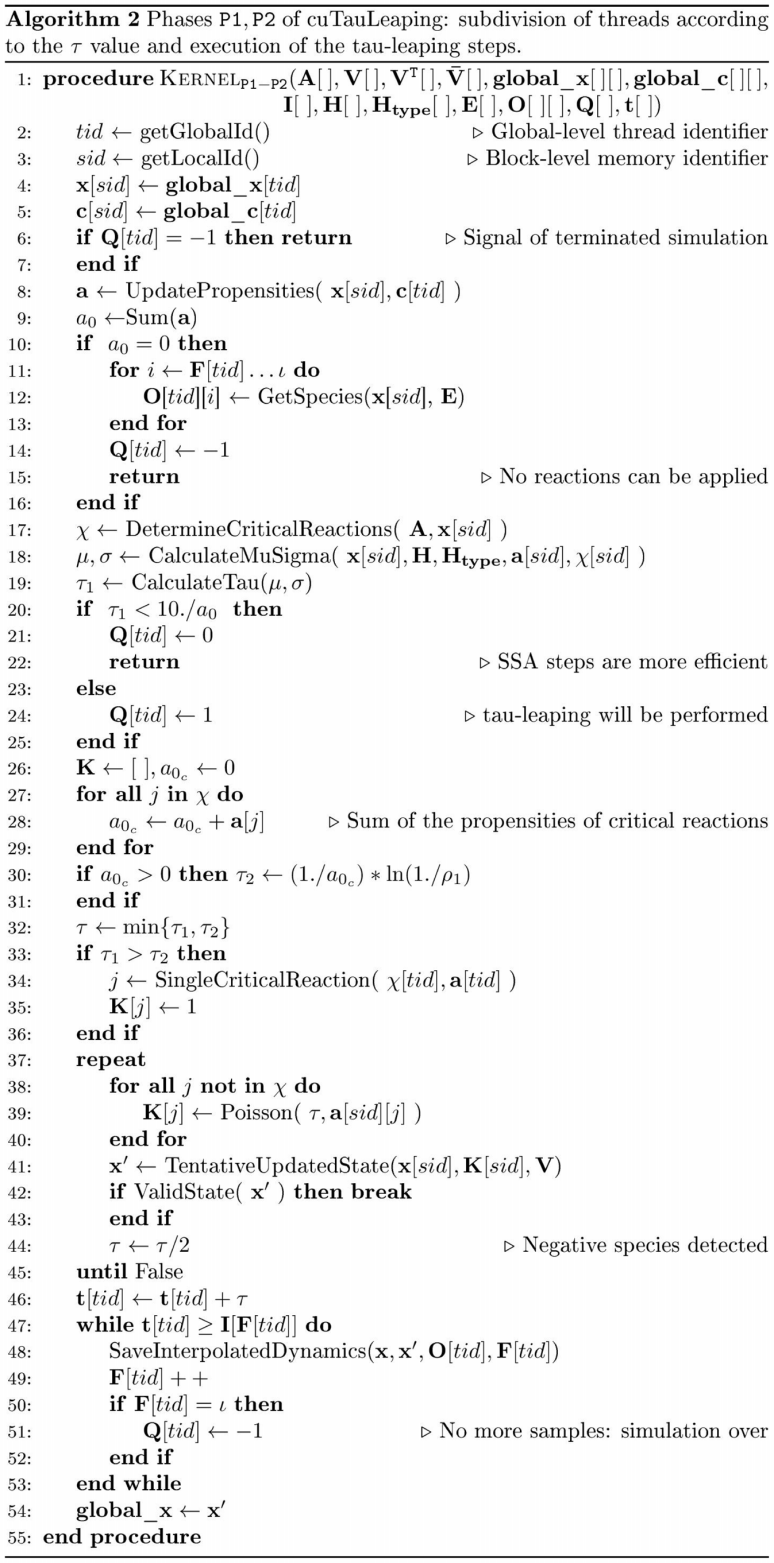
Pseudocode of cuTauLeaping – kernel 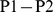. Device-side pseudocode of kernel 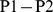 in cuTauLeaping, implementing the subdivision of threads according to the 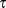 value and the execution of a tau-leaping step. The kernel starts by loading the vectors 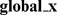 and 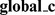 – which correspond to the current state of the system and to the values of stochastic constants, respectively – from the global memory areas that contain these data for all threads. Since these information are frequently accessed, they are immediately copied into the faster shared memory as vectors **x** and **c**, respectively. The kernel continues by verifying that the 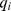 value for the running thread 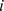 is not equal to the signal of terminated execution (i.e., 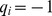). Then, it calculates the propensity functions of all reactions and accumulates their values in 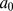; if 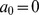, the remaining time instants where the dynamics of the system is sampled are set to the current state and the simulation is terminated. The kernel concludes the phase P1 by calculating a putative 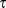 value for the tau-leaping step: if 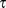 is smaller than 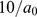, then thread 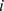 is halted and 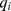 is set to 0, so that it will perform the SSA steps during the next phase. Otherwise, the tau-leaping algorithm is performed by executing a set of non-critical reactions and (possibly) one critical reaction and, if the simulation has overrun one of the sampling time instants, the state stored in 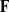 is determined by linear interpolation.

**Figure 5:**
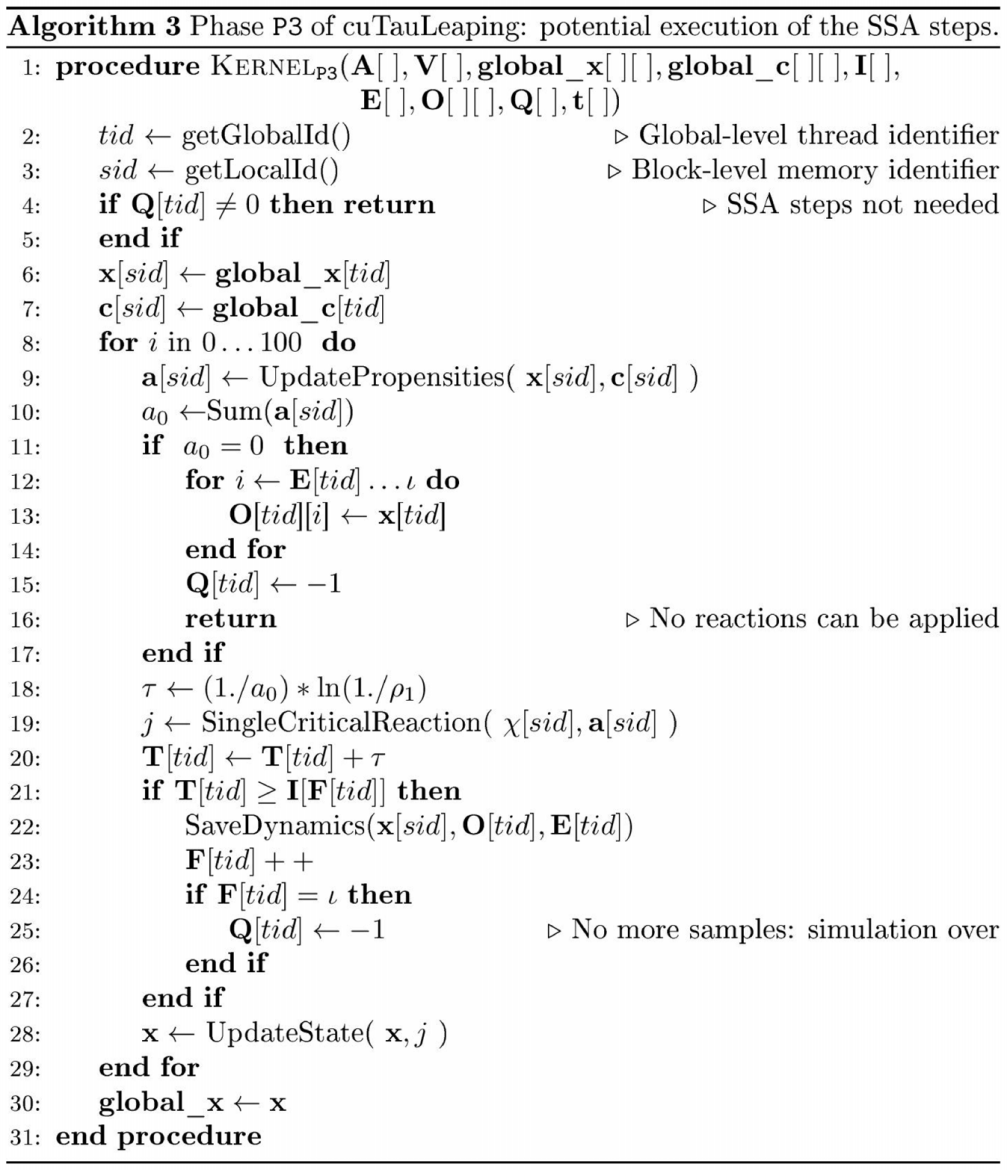
Pseudocode of cuTauLeaping – kernel 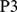. Device-side pseudocode of kernel 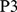 in cuTauLeaping, implementing the execution of the SSA steps. The kernel starts by loading the vectors 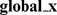 and 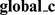 – which correspond to the current state of the system and to the values of stochastic constants, respectively – from the global memory areas that contain these data for all threads. Since these information are frequently accessed, they are immediately copied into the faster shared memory as vectors **x** and **c**, respectively. The kernel continues by verifying that the 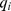 value for the running thread 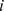 is equal to the signal corresponding to SSA (i.e., 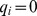). Then, it performs a fixed number of SSA steps (100 in our default setting), **where a single reaction is executed at each step**, storing the system state at the sampled time instants 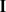.

**Figure 6:**
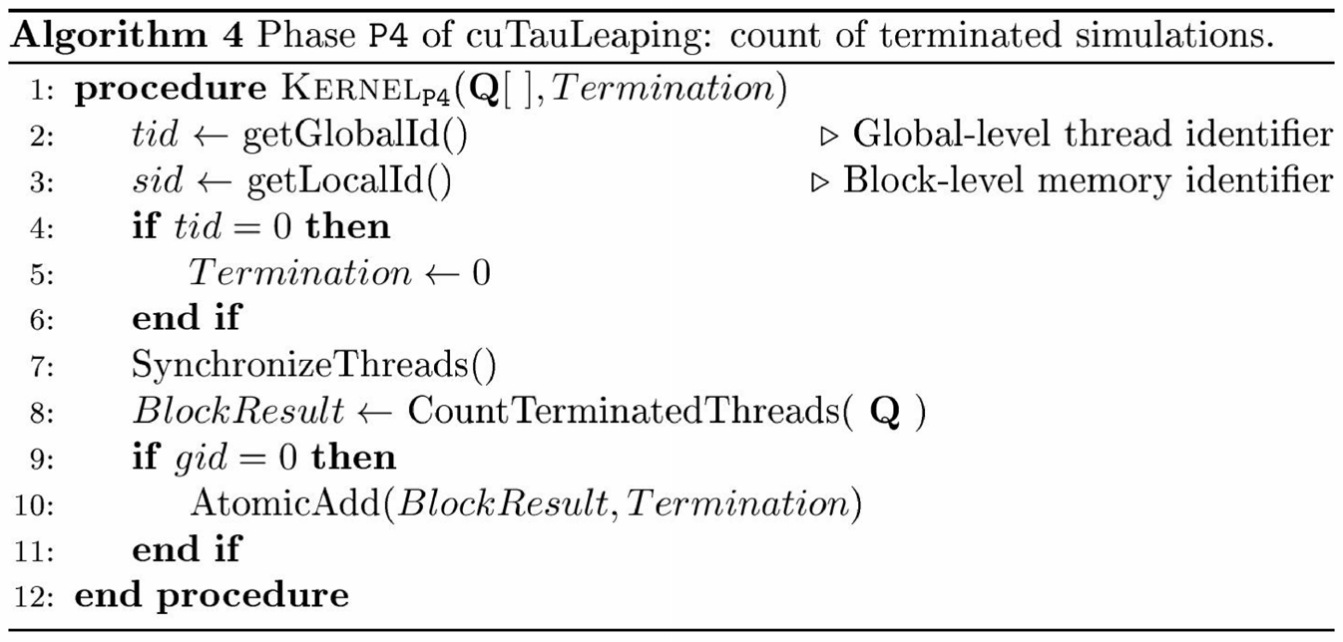
Pseudocode of cuTauLeaping – kernel 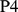. Device-side pseudocode of kernel 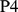 in cuTauLeaping, implementing the verification of the termination of all simulations. The verification is performed by means of CUDA's hardware accelerated synchronization and counting features, which allow to count the threads of a block which satisfy a specific predicate. By exploiting CUDA's atomic functions, we accumulate the total number of threads which satisfy the predicate 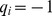: if it is equal to the number of threads, the execution of all parallel simulations is completed.

In Figure 3 we report the pseudocode of the host side procedure devoted to invoke the CUDA kernels; in Figures 4, 5, 6 we present the pseudocodes of kernels 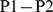, 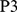, 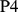, respectively. Kernels are iteratively repeated until 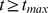 for all threads. This termination criterion is efficiently verified by kernel 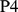 that exploits two advanced CUDA functionalities introduced with the Fermi architecture: *synchronizations with predicate evaluation* and *atomic functions*. Synchronization functions are generally used to coordinate the communication between threads, but CUDA allows to exploit these functions to evaluate a predicate for all threads in a block; atomic functions allow to perform read-modify-write operations without any interference from any other thread, therefore avoiding the race condition. A combination of these functionalities allows to determine whether all threads have terminated their execution (i.e., the predicate is 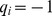, for all 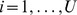). In addition, since both functionalities are hardware-accelerated, the resulting computational complexity is 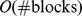, making them more efficient than other equivalent methodologies, e.g., parallel reduction [67], whose complexity is 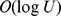 (note that, in general, 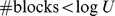).

**Figure 7:**
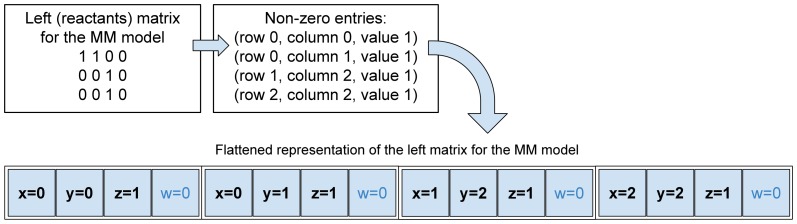
Schematization of the flattened representation of the stoichiometric information. The stoichiometry of chemical reactions is generally represented by (usually sparse) matrices, corresponding to the variation of the species appearing either as reactants or products; however, both tau-leaping and SSA exploit only the non-zero values of these matrices. Each stoichiometric matrix can be pre-processed to identify its non-zero values and discard the remaining ones, thus reducing the number of reading operations required by the two stochastic algorithms. Our strategy to reduce the size of these matrices consists in flattening each matrix as a vector of triples (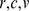), where 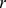 is the row index, 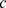 is the column index and 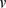 is the non-zero value in 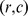. In our implementation, both 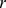 and 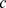 indices are 0-based and triples are stored using vectors of CUDA's 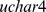 data types, that have the advantage of requiring a single instruction to fetch an entry. The top part of this figure shows the values appearing in the 3×4 stoichiometric matrix of reactant species of the Michaelis-Menten model (MM), which consists of 3 reactions over 4 molecular species (see Text S1). Note that only four cells of this matrix have non-zero values; the bottom part of the figure shows the corresponding 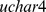 vector.

In order to further improve the performance of the simulation execution, it is better not to code the stoichiometric information by means of matrices. In cuTauLeaping, we flattened the stoichiometric matrices 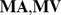, 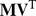 and 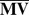 – which are typically sparse matrices – by packing their non-zero elements into arrays of CUDA vector types, named 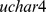, whose components are accessed by means of 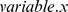, 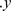, 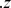, and 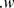; the vectors corresponding to these matrices are named 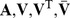, respectively. Since both 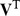 and 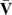 vectors can assume negative numbers, we use an offset to store their 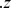 values as *unsigned chars*, and subtract the offset during the calculations on the GPU to yield back the correct negative numbers. An example of this implementation strategy is shown in Figure 7 which schematizes, for the Michaelis-Menten model, the conversion of the matrix 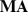 into the corresponding flattened representation 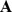. By using this strategy, the complexity of the calculations needed for both SSA and tau-leaping decreases from 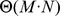 to 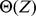, where 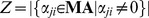 is the number of non-zero entries in 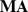. For each non-zero entry, we store into the 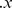 and 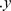 components the corresponding row and column indices of 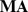, respectively; the 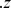 component is used to store the stoichiometric value. Note that, even though the 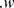 component is left unused, it is more efficient to employ the 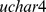 vector type rather than 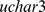, because the former is 4-aligned and takes a single instruction to fetch the whole entry, while the latter is 1-aligned, and would require three memory operations to read each entry of the flattened vector. It is worth noting that the use of an *unsigned char* data type implies that cuTauLeaping could deal with models with up to 256 reactions and molecular species; for larger systems, data types with greater size must be exploited. Anyway, the maximum size of a model is also limited by the shared memory available on the GPU; we provide a detailed analysis of this issue in the Discussion section.

cuTauLeaping is also optimized for what concerns the calculation of 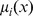 and 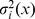 values (Equation 4). These values are related to each species 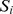, and represent an estimate of the change of the propensity functions, based on all possible reactions in which the species 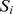 is involved. For this reason, the flattened representation of the matrix 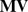 cannot be exploited here; therefore, to obtain an efficient calculation of these values, we introduced the flattened transposed stoichiometric matrix 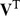.

In order to further increase the performances of cuTauLeaping, we also optimized the CUDA code to better exploit the GPU architecture. The first optimization consists in keeping the register pressure low, in order to avoid the *register spilling* into global memory and to increase the occupancy of the GPU. This is achieved by partitioning the tau-leaping algorithm into multiple kernels, allowing a strong reduction of the consumption of hardware resources (i.e., the register pressure). This CUDA optimization technique, known as *branch splitting*, was shown to achieve a relevant gain in performances [68].

Another typical optimization of CUDA is to ensure coalesced access to data, i.e., an aligned and sequential organization of the memory, for all data structures that are updated by each thread. In our implementation we granted coalescence to all data structures that are private to each thread, that is, the system state 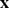, the stochastic constants 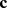, and so on. However, there is some shared information that is not inherently coalesced:

- the stoichiometric flattened vectors 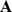 and 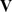 (used by kernel 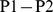 and kernel 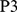), 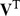 and 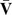 (used by kernel 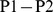);
- the vector 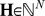 (used by kernel 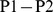), containing the highest order of all reactions in which each molecular species appears as a reactant;
- the vector 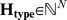 (used by kernel 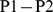), containing the information about the maximum number of reactant molecules involved in the highest-order reactions.

The data structures used to store the stoichiometric information (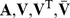) are not modified during the simulation and are common to all threads, and can be conveniently loaded into the constant memory. This peculiar CUDA memory is immutable and cached, so that the uncoalesced access pattern does not have any impact on the performances.

Note that, in contrast to other GPU implementations of tau-leaping [39], cuTauLeaping exploits the vector 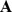 to correctly evaluate the propensity functions of the reactions whose reactant species appear also as products in the same reaction: in cases like this, the net balance of the consumed and produced chemicals that is stored in vector 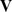 would not carry sufficient information to distinguish between reactants and products. For instance, given 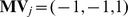, it is impossible to establish whether this state change vector corresponds to a reaction of the form 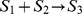 or 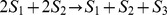; on the contrary, the information stored in 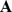 allows to discriminate between the two cases.


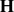
 and 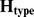 vectors are necessary to evaluate the length of the step 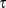 (Equation 3), which is determined at each step according to the current state of the system; 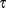 is then exploited to update the simulation time of each thread, denoted by 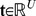. Both 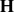 and 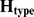 vectors, anyway, can be calculated offline by preprocessing the stoichiometric matrices 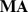 and 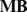 while they are loaded.

In addition, both kernels 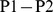 and 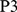 exploit the following three vectors:

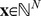
 is the current state of the system;

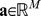
 contains the values of the propensity functions of the reactions;

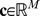
 contains the values of the stochastic constants.

Kernel 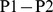 exploits four additional vectors:

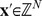
 is the putative state of the system (note that the elements of this vector might assume negative values);

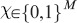
 contains the information about critical reactions, stored as 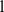 s, and non-critical reactions, stored as 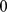 s;

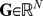
 contains the auxiliary values used to calculate the length of the time step satisfying the leap condition, which are computed by using the vectors 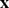, 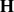 and 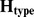 (see details in [27]);

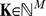
 contains the samples of the Poisson distributions corresponding to the number of times each reaction will be fired in the current step.

These vectors are coalesced, but frequently exploited by tau-leaping and SSA. In order to minimize the latencies due to the frequent access to the global memory, for each thread we allocate 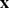, 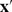, 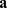, 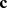, 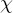 and 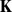 into the shared memory. Being an on-chip memory, latencies of the shared memory are about two orders of magnitude lower than that of the global memory; the use of shared memory allows a reduction of the global bandwidth usage [69] and provides a relevant performance boost. In contrast, we memorize 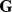 into the global memory, since its values are used only twice during the simulation step to determine the 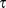 value. Since cuTauLeaping was specifically designed to be embedded into other applications – in particular, the computational tools for PE, PSA and RE that we previously developed [6], [7], [35], which rely on the execution of a large number of simulations – we also copy the stochastic constants into the shared memory vector 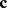.

To obtain a more efficient implementation, an additional strategy consisted in restructuring the tau-leaping algorithm in order to avoid the conditional branches as much as possible. In cuTauLeaping, branches were removed by unrolling loops and by allowing redundant calculations in favor of a uniform control flow.

There are two more relevant facets of cuTauLeaping implementation: the storage of the simulated dynamics and the “feed” of molecular species amounts. The storage of the entire temporal evolution of all species, associated to each thread on the GPU, cannot be realized, since we cannot determine *a priori* how many steps each simulation will take. Indeed, whenever a kernel is launched, the required amount of memory must be statically pre-allocated from the host; it is therefore fundamental to set the number of time instants in which the dynamics is sampled, before each simulation starts. Moreover, the naïve storage of all molecular species, even in the case of a few sampled time instants, may be unfeasible. For instance, in the case of the Ras/cAMP/PKA pathway, storing the dynamics of 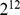 simulations, considering 1000 samples stored as single-precision floating point values, would require 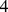
(bytes)
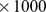
(samples)
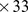
(species)
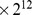
(simulations)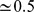 GB, which is very close to the memory amount of an average GPU. Therefore, we make use of four additional global memory vectors:

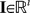
 contains the 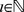 time instants in which the temporal evolution of the system is stored;

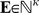
 contains the indices of the 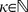 molecular species whose amounts are stored (for some 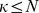);

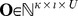
 contains the 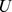 simulated dynamics of the molecular species in 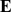, sampled during the time instants in 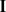;

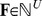
 stores, for each thread, the pointer to the next time instant in 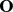, i.e., when the 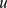-th simulation reaches the 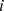-th sampling time instant, 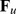 is set to 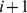.

Finally, in order to fully exploit the SM cache, also the values of 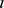, 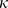, 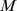, 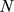, 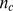, 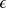, 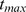 and the size of the flattened vectors 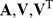 and 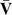, are stored as constants into the constant memory of the GPU.

### Computational results

In this section we present a comparison of the computational effort of GPU and CPU for the simulation of four stochastic models of increasing size and complexity: the Michaelis-Menten kinetics (MM) [40], a prokaryotic gene regulatory network (PGN) [35], [41], the Schlögl model [70], [71] and the Ras/cAMP/PKA signal transduction pathway in yeast [6], [43], [44]. The definition of each model, as well as the values of the initial molecular amounts and of the stochastic constants used to run simulations, are given in Text S1.

To analyze the performances of cuTauLeaping, the same simulations executed on GPU were carried out on a CPU architecture by exploiting the tau-leaping algorithm implemented in the software COPASI (version 4.8 Build 35, running on Windows 7 64-bit) [66]. COPASI has been recently integrated with a server-side tool, named Condor [33], that handles COPASI jobs, automatically splits them in sub-jobs and distributes the calculations on a cluster of heterogeneous machines; in the present work we do not use this possibility as we are interested in COPASI as a single-node CPU-bound reference implementation, which is currently single-threaded and does not exploit the physical and logical cores of the CPU.

The GPU used for the tests is a Nvidia GeForce GTX 590, a dual-GPU video card equipped with 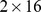 SMs for a total of 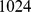 cores (cuTauLeaping automatically distributes the workload on the available SMs); the performances were compared with a quad-core CPU Intel Core i7-2600 with a clock rate of 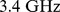.

In all simulations, the total simulation time 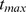 for MM, PGN and Schlögl models was set to 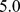, 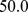 and 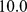 a.u., respectively, while for the Ras/cAMP/PKA model it was set to 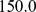 a.u.; for each simulation, we stored 100 samples of all the molecular species occurring in the system. The value of the error control parameter of tau-leaping was set to 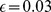, as suggested in [27].

**Figure 8:**
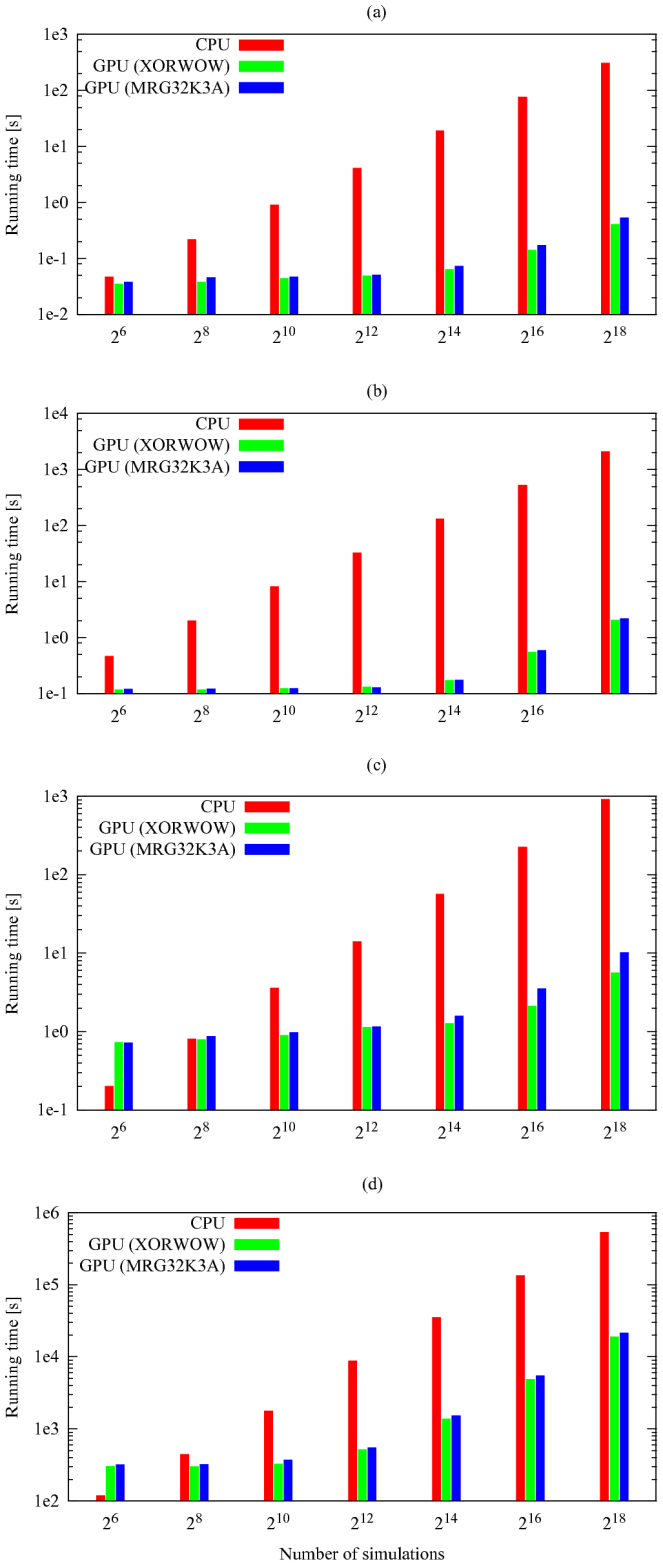
Comparison of the computational time of CPU tau-leaping and cuTauLeaping. Comparison between the computational time taken by cuTauLeaping and COPASI CPU tau-leaping to execute different batches of stochastic simulations of the Michaelis-Menten (MM) model (**a**), the Prokaryotic Gene Network (PGN) model (**b**), the Schlögl model (**c**), and the Ras/cAMP/PKA pathway (**d**) (see Text S1 for models definitions). For each model, cuTauLeaping becomes more profitable than the CPU counterpart when a certain number of parallel simulations is run, with a break-even that depends on the complexity of the system: for the MM and PGN models, cuTauLeaping is more effective when around 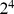 parallel simulations are run, while for the Schlögl model and the Ras/cAMP/PKA pathway the break-even is around 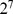 simulations. Considering the speedup, the best results achieved with cuTauLeaping – with respect to COPASI – are around 583× for the MM model, 961× for the PGN model, 90× for the Schlögl model, and 25× for the model of the Ras/cAMP/PKA pathway (see also Table 1).

The results of the comparison between cuTauLeaping and COPASI CPU tau-leaping are summarized in Table 1, which reports the running time (in seconds) obtained by executing different batches of simulations of each model; these results were obtained using the RNG MRG32K3A in cuTauLeaping. Table 1 clearly show the advantage of cuTauLeaping as the number of simulations increases. Interestingly, because of the architectural differences and the different clock rates, a single run of cuTauLeaping may be slower than the CPU counterpart, and it becomes fully profitable only by running multiple simulations. For instance, in the case of the Ras/cAMP/PKA model (Figure 8d), the break-even is reached around 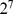 simulations. Thus, when less than 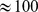 simulations of this specific pathway are needed, the use of a CPU implementation may be more convenient. Nonetheless, statistical analyses of stochastic temporal evolutions of biological systems require large batches of simulations (usually 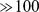) to derive statistically significant measures of the analyzed system dynamics. Note that, in general, the analytical determination of the break-even for an arbitrary model is a hard task, because it depends on its size (the number of reactions and molecular species) as well as on its parameterization that might lead to stiffness phenomena, able to affect the running time of the used simulation algorithm. Moreover, if a biological system is characterized by multistability or very large fluctuations in the dynamics of some molecular species (e.g., those occurring in low amounts), simulations running in different threads can be characterized by a high divergence of the execution flow, thus resulting in reduced parallelism and, consequently, in worse performances. For instance, if two threads simulating the same system reach very different system states, they can take different branches within the code (e.g., due to a rejection of an invalid putative state 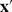 of the system), an event that can greatly affect the performance of the GPU.

**Table 1:** Comparison of computational time of COPASI CPU tau-leaping and cuTauLeaping.

*Model*	*Simulations*	*CPU time*	*GPU time* *	*Speedup*
Michaelis-Menten	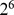	0.047	0.038	1.23×
	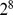	0.219	0.046	4.75×
	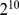	0.905	0.047	19.25×
	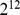	4.087	0.051	80.14×
	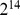	19.001	0.073	260.29×
	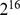	76.691	0.172	445.87×
	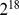	309.241	0.530	583.47×
Prokaryotic gene network	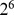	0.468	0.120	3.90×
	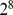	1.997	0.121	16.50×
	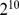	8.112	0.124	65.42×
	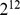	32.807	0.128	256.30×
	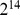	130.885	0.175	747.91×
	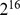	526.535	0.591	890.92×
	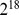	2095.48	2.18	961.23×
Schlögl	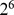	0.202	0.723	0.28×
	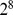	0.811	0.875	0.92×
	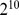	3.603	0.979	3.68×
	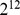	13.993	1.156	12.10×
	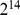	56.254	1.578	35.64×
	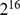	224.454	3.534	63.51×
	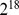	905.664	10.163	89.11×
Ras/cAMP/PKA	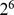	118.873	320.1	0.37×
	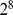	445.632	322.4	1.38×
	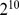	1769.58	372.4	4.75×
	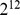	8828.05	551.4	16.01×
	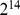	35027.9	1530.1	22.89×
	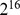	133733	5482	24.39×
	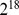	534932**	21470	24.92×

*Exploiting the MRG32K3A random numbers generator.

**Estimated value.

For the sake of completeness, we also plot in Figure 8 the running times of cuTauLeaping obtained by using both RNGs available in cuTauLeaping, XORWOW and MRG32K3A, and compare these results with the running time of COPASI CPU tau-leaping (see also Table 2 in Text S2). To fully compare these two RNGs, in Text S2 we show an example of the frequency distribution of the amount of the molecular species 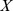 in the Schlögl model, obtained executing different batches of parallel simulations using either MRG32K3A or XORWOW, and present the results of the Kolmogorov-Smirnov test. Altogether, our results show that, despite XORWOW presents statistical flaws and therefore should not be safely exploited to carry out Monte Carlo simulations, the frequency distributions of 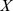 obtained with XORWOW and MRG32K3A can be considered equivalent; in addition, XORWOW allows to achieve a higher speedup with respect to MRG32K3A.

Finally, in order to investigate the influence of the size of the simulated system on cuTauLeaping performances, we executed several tests on randomly generated synthetic models (RGSM). In particular, we analyzed six distinct parameterizations of 100 different RGSM, each one consisting of 35 reactions and 33 species. The rationale behind the choice of this model size and various initial conditions was to compare the computational costs of cuTauLeaping for the simulation of RGSM on the one side, and the Ras/cAMP/PKA model on the other side. RGSM were generated according to the methodology proposed by Komarov *et al.* [39], and following two different strategies for the selection of the values of the stochastic constants. The results of these tests are given in Table 2 where, for each synthetic model, the average running times (given in seconds) were evaluated by executing 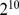 parallel simulations with 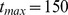 a.u., to allow a direct comparison with the results listed in Table 1.

**Table 2:** Running times of cuTauLeaping for the simulation of randomly generated synthetic models.

*Test n.*	*Model size*	*Initial molecular amounts*	*Stochastic constants interval*	*Average running time (standard deviation)*
1	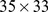	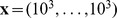	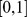	1.906 (1.396)
2	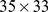	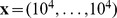	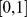	8.149 (7.866)
3	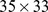	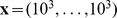	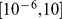 *	1.436 (0.7)
4	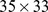	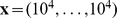	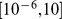 *	8.263 (6.053)
5	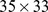	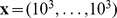	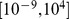 *	1.247 (0.543)
6	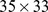	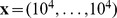	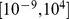 *	18.509 (91.976)
7	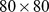	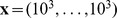	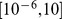 *	13.242 (10.623)
8	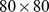	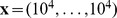	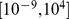 *	96.133 (302.05)

*Logarithmic sampling.

In tests 1 and 2 we randomly selected the values of stochastic constants of the RGSM with a uniform probability in 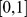; the initial molecular amounts were set to 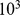 (test 1) and 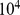 (test 2) for all species appearing in the systems. Both tests show that, on average, the computational time required for 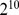 parallel simulations of RGSM is much lower than the time needed for the same number of parallel simulations of the Ras/cAMP/PKA model. In addition, we observe that the initial molecular amounts considered in the parameterizations actually influence the results; in general, higher quantities lead to higher average running times.

In tests 3 to 6 we exploited a modified strategy to select the values of stochastic constants, which were logarithmically sampled in the given range in order to uniformly span over different orders of magnitude. To obtain more realistic parameterizations, in tests 3 and 4 this range was defined according to the smallest and to the highest values of stochastic constants appearing in the Ras/cAMP/PKA model (see Table 6 in Text S1); in tests 5 and 6, the range was widened to six orders of magnitude larger than the values used in tests 3 and 4. Also in these cases, the average running time required for the simulation of the RGSM is lower than the time needed to execute the same number of parallel simulations of the Ras/cAMP/PKA model. Moreover, we observe that the running time is mainly influenced by the initial molecular amounts rather than the values of the stochastic constants used in the different parameterizations.

In addition, we carried out 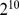 parallel stochastic simulations of RGSM whose sizes are approximately 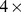 bigger than the Ras/cAMP/PKA model (tests 7 and 8). Even considering the worst result (test 8), the average running time required to simulate these larger synthetic models is still low, suggesting that the system size has not a direct impact on the performances of cuTauLeaping, while the complexity of the system – such as the presence of positive or negative feedbacks possibly leading to oscillatory dynamics (as in the Ras/cAMP/PKA model), or reactions that lead to stiffness (as in the Schlögl model) – is much more relevant.

### The analysis of bistability in the Schlögl model

Bistability is a capacity exhibited by many biological systems, consisting in the possibility of switching between two different stable steady states in response to some chemical signaling (see, e.g., [72]–[74] and references therein). The Schlögl model is one of the simplest prototypes of chemical systems presenting a bistable dynamic behavior [70], [71]. This system is characterized by the fact that, starting from the same initial conditions, its dynamics can reach either the low or the high steady state; switches between the two steady states can also occur due to stochastic fluctuations.

cuTauLeaping can be efficiently exploited for the execution of a massive number of simulations of the Schlögl model, with the same initial parameterization, in order to produce a frequency distribution of the molecular species that exhibits the bistable behavior. Generally speaking, this kind of investigation allows the implicit identification of attractors and multiple steady states of a system, and helps to (empirically) determine the probability of reaching a particular state during the dynamical evolution of the system itself.

**Figure 9:**
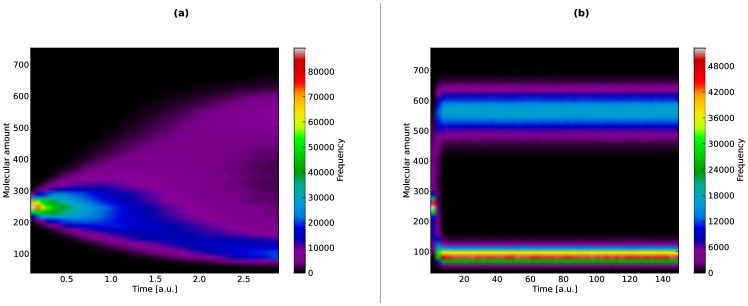
Frequency distribution of bistable states in the Schlögl model. Frequency distribution of the molecular amount of molecular species 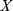 in the Schlögl model, calculated using a total of 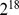 parallel simulations executed by cuTauLeaping. (**a**) Plot of the frequency distribution of 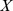 considering 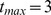 a.u., to detect the bistable switching behavior that takes place in the first time instants of the dynamics; a slightly higher probability to reach the low steady state can be observed, starting from the initial state of the Schlögl system (described in Text S1). (**b**) Plot of the frequency distribution of 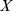 considering 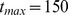 a.u., to investigate the stability of the two steady states of the system; the heatmap highlights the two stable states (around 100 and 600 molecules of species 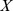), and shows larger stochastic fluctuations around the high steady state.

To this aim, we performed 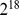 simulations of the Schlögl model, keeping track of the molecular amount of species 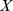 in each simulation, sampled in 100 time instants uniformly distributed over the simulation time. In particular, to detect the initial jump either to the low or to the high steady states, that takes place in the first time instants of the simulations, we performed 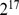 simulations with 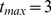 a.u.; then, for a deeper investigation of the bistable switching behavior of the system, the other simulations were executed considering 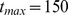 a.u.. We used the results of the simulations to calculate the histograms of the molecular amount of 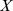, that were then exploited to realize a heatmap showing the frequency distribution of this species between the two stable steady states. In Figure 9a we show the initial transient of the dynamics, where a slightly higher probability to reach the low steady state can be observed, starting from the initial configuration of the Schlögl system (described in Tables 4 and 5 in Text S1). Figure 9b shows the frequency distribution of reaching either the low or the high steady state (around 100 and 600 molecules of species 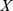, respectively), highlighting a larger variance concerning the high steady state.

**Figure 10:**
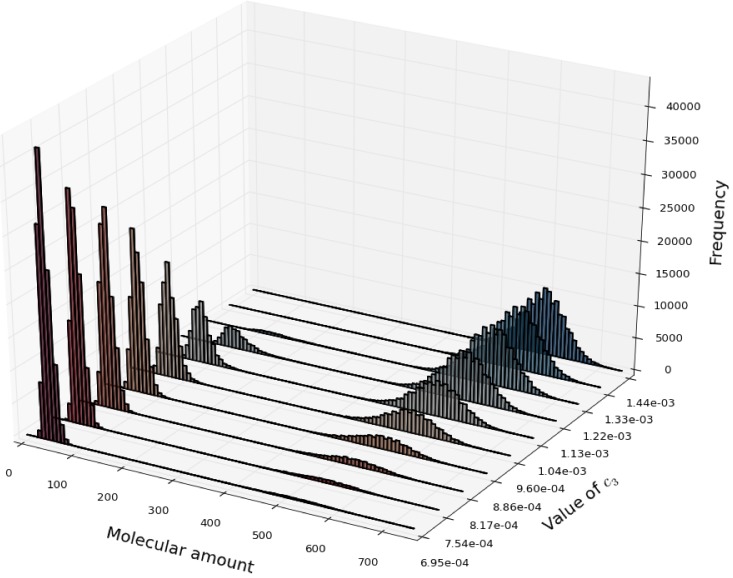
Parameter sweep analysis of the Schlögl model. Results of a PSA-1D on the Schlögl model, in which the value of the stochastic constant 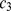 is varied in the interval 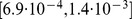 (the set of reactions and the values of all other parameters are given in Tables 4 and 5 in Text S1). Each frequency distribution is calculated according to 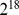 simulations executed by cuTauLeaping, measuring the amount of the molecular species 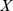 at the time instant 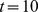 a.u., considering ten different values of the stochastic constant 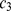 within the sweep interval. The figure shows that increasing values of 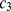 induce a decrease (increment) in the frequency distribution of 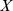 concerning the low (high) steady state, with intermediate values of 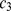 characterized by an effective bistable behavior.

In Figure 10, we show the frequency distribution of molecular amounts of 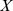 in perturbed conditions of the Schlögl model, evaluated by exploiting a PSA-1D in which the value of the stochastic constant 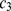 is uniformly varied in the interval 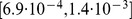. The frequency distribution was calculated according to 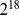 simulations, where the dynamics was sampled at the single time instant 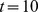 a.u., according to ten different values of the stochastic constant 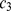 in the chosen sweep range. The total running time to execute this PSA-1D was 34.92 seconds with cuTauLeaping, and 1013.51 seconds with COPASI, thus achieving a 116× speedup. Figure 10 shows that increasing values of 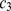 induce a decrease (increment, respectively) in the frequency distribution of 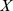 concerning the low (high, respectively) steady state, whereas for intermediate values of 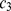 the system is characterized by an effective bistable behavior.

**Figure 11:**
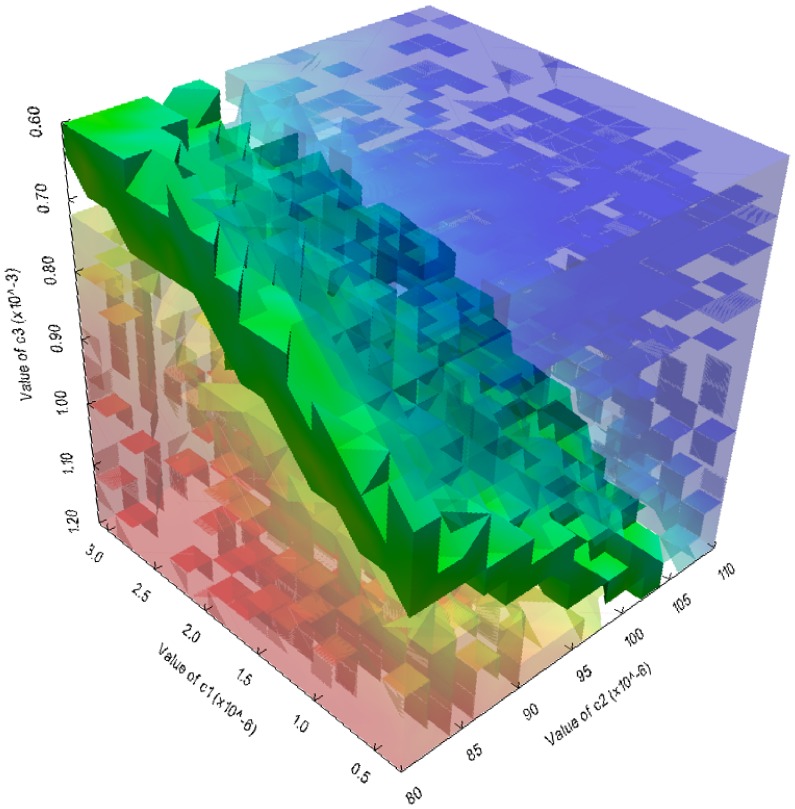
Three-dimensional parameter sweep analysis of the Schlögl model. Results of a PSA-3D on the Schlögl model, performed by varying the stochastic constants 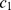, 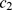 and 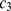 in the intervals 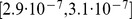, 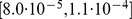 and 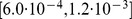, respectively. The values of the stochastic constants were uniformly sampled in a 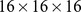 three-dimensional lattice; for each sample, we executed 256 simulations with cuTauLeaping (for a total of 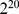 simulations) and evaluated the frequency distribution of the amount of the molecular species 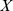 at the time instant 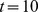 a.u.. This set of values was then partitioned according to the reached (low or high) stable steady state; in the plot, the red (blue) region corresponds to the parameterizations of the model which yield the high (low) steady state most frequently. The green region represents a set of conditions whereby both steady states are equally reached.

Finally, in Figure 11 we show the results of a PSA-3D performed by simultaneously varying the values of the stochastic constants 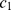, 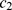 and 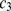 in the ranges 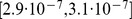, 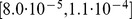 and 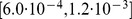, respectively; for each stochastic constant, taken independently from the others, the chosen range corresponds to a condition of effective bistability of the Schlögl model. The values of the three stochastic constants were uniformly sampled in a 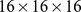 three-dimensional lattice; for each sample, we executed 256 simulations (for a total of 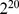 simulations) and evaluated the frequency distribution of the amount of species 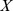 at the time instant 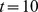 a.u.. This set of values was then partitioned according to the reached (low or high) stable steady state; in Figure 11, the red (blue, respectively) region corresponds to the parameterizations of the model which yield the high (low, respectively) steady state most frequently. The green region represents a set of conditions whereby both steady states are equally reached.

### Parameter sweep analysis of the Ras/cAMP/PKA model

In this section we present the results of a PSA carried out on the stochastic model of the Ras/cAMP/PKA pathway [6], [44]. In *S. cerevisiae*, this pathway plays a major role in the regulation of metabolism, in stress resistance and to control the cell cycle progression [75], [76]. In [6], it was shown that intrinsic noise within the Ras/cAMP/PKA pathway can enhance the robustness of the system in response to different perturbations of the model parameters, ensuring the presence of stable oscillatory regimes, as previously investigated for other biological systems (see [77] and references therein). Indeed, stochastic simulations of the functioning of this pathway showed that yeast cells might be able to respond appropriately to an alteration of some basic components – such as the intracellular amount of pivotal proteins, that can be related to the stress level [78], [79] – fostering the maintenance of stable oscillations during the signal propagation. This behavior might suggest a stronger adaptation capability of yeast cells to various environmental stimuli or endogenous variations.

In [6], [44], in particular, we showed that the intracellular pool of guanine nucleotides (GTP, GDP), as well as the molecular amounts of protein Cdc25 – that positively regulates the activation of Ras protein, and that is negatively regulated by PKA – are both able to govern the establishment of oscillatory regimes in the dynamics of the second messengers cAMP and of protein PKA. In turn, this behavior can influence the dynamics of downstream targets of PKA, such as the periodic nucleocytoplasmic shuttling of the transcription factor Msn2 [78], [80]. In addition, in [6], [44] we highlighted that stochastic and deterministic simulations of the Ras/cAMP/PKA pathway can yield qualitatively different outcomes: in some conditions, characterized by very low amounts of pivotal proteins in this pathway (e.g., Cdc25), the stochastic approach provides stable oscillatory regimes of cAMP, while the deterministic approach shows damped oscillations. Therefore, these results remark the role played by noise in the Ras/cAMP/PKA pathway and the usefulness of executing stochastic simulations.

To deeply investigate the role played by guanine nucleotides and Cdc25, in this work we further analyzed the extended version of the Ras/cAMP/PKA model presented in [6], [44], where the reactions responsible for the occurrence of oscillatory behaviors were included. To this aim, we performed a PSA-2D to simulate the system dynamics in perturbed conditions, where we simultaneously varied the amount of GTP in the interval 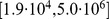 molecules (corresponding to a reduced nutrient availability, up to a normal growth condition) and the amount of Cdc25 in the interval 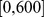 molecules (ranging from the deletion to a 2-fold overexpression of these regulatory proteins). A total of 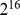 different initial parameterizations were uniformly distributed over this bidimensional parameter space.

**Figure 12:**
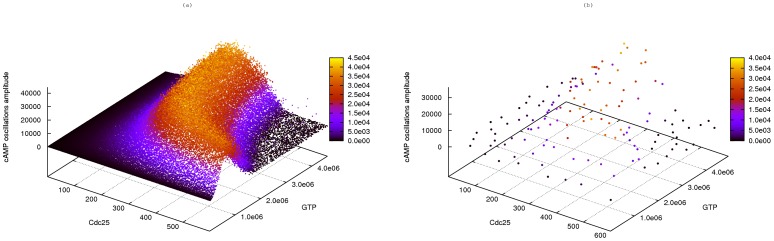
Bidimensional parameter sweep analysis of the Ras/cAMP/PKA model. Results of a PSA-2D on the Ras/cAMP/PKA model by varying the amount of GTP in the interval 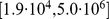 molecules (ranging from a reduced nutrient availability to a normal growth condition), and the amount of Cdc25 in the interval 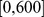 molecules (ranging from the deletion to a 2-fold overexpression of this GEF proteins). The figure shows the amplitude of cAMP oscillations, evaluated as described in [44]; an amplitude value equal to zero corresponds to a non oscillating dynamics. (**a**) Plot of the results obtained by running 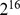 parallel simulations with cuTauLeaping; (**b**) plot of the results obtained by running 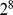 sequential simulations, performed on the CPU. The two batches of parallel and sequential simulations were executed with a comparable computational time.

In Figure 12a we plot the amplitude of cAMP oscillations in each of these initial conditions, where an amplitude value equal to zero corresponds to a non oscillating dynamics; the amplitude values of cAMP oscillations were calculated as described in [44]. This figure shows that oscillatory regimes are established for basically any value of GTP when the amount of Cdc25 is at normal condition or slightly lower, while if the amount of Cdc25 increases, no oscillations of cAMP occur when GTP is high, but oscillatory regimes are still present if GTP is low. In order to compare the advantage of using cuTauLeaping to perform this stochastic analysis with respect to a CPU implementation, in Figure 12b we present the previous analysis performed on the CPU [6], which was obtained with a comparable computational time, albeit in the case of the CPU-based analysis only 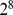 parameterizations of the Ras/cAMP/PKA pathway (corresponding to 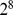 independent simulations) could be analyzed.

These computational results can suggest possible interesting behaviors of the biological system under investigation. In this case, for instance, the establishment of oscillatory regimes in the above mentioned conditions can be due to the fact that Ras proteins are more frequently in their inactive state (that is, loaded with GDP instead of GTP) when the ratio GTP/GDP decreases. Since in normal growth conditions the concentration of GTP is 3 to 5 times higher than GDP, the decreased activity of Ras proteins in the considered perturbed conditions – which are characterized by a favored unproductive binding/unbinding with GDP – can induce the establishment of an oscillatory regime (see also [6] for more details).

## Discussion

To reduce the computational costs related to the analyses of mathematical models of real biological systems, two conceptually simple ways can be considered to parallelize stochastic simulations. The easiest solution consists in generating multiple threads on multi-core workstations, but it immediately turns out to be undersized, since the number of cores on high-end machines can be far lower than the number of simulations required for computational analysis as PE, PSA, SA and RE. The other way consists in distributing the stochastic simulations on a cluster of machines, which may as well result inadequate for several problems. First of all, it is economically expensive and very power-demanding; secondly, it takes a dedicated software infrastructure to handle workload balancing, network communication and the possible errors due to nodes downtime or server-node communication issues; thirdly, if the nodes of the cluster are heterogeneous, the slowest machines may represent a bottleneck for the whole task. In addition, a cluster implementation may not always scale well because of two problems: on the one hand, the speedup is approximately proportional to the number of independent simulations that run on a dedicated node (i.e., a million nodes for common tasks like PE and SA); on the other hand, the running time of each simulation can be larger than the overhead requested for server-node communication.

An alternative methodology to perform multiple and massively parallel simulations consists in exploiting the GPGPU architecture. The modern GPU of mid-range price contains thousands of cores that – as long as the computational task can be subdivided and optimized for a SIMD architecture – allow an impressive peak of computational power, and also a higher energetic efficiency with respect to an equivalent CPU-based solution.

Taking into account all these aspects, we developed cuTauLeaping, an implementation of tau-leaping algorithm as a set of strongly optimized CUDA kernels, able to simultaneously execute multiple independent simulations on a single machine. The specific design of cuTauLeaping presents some additional advantages: it avoids any memory transfer to and from the host, thus reducing the overall running time, and it can be embedded into the GPU-based software framework that we developed for PE [35], [81] and RE [7]. As a matter of fact, the modularity of our implementation and the mutual independence of the multiple simulations allows to easily wrap cuTauLeaping with any other methodology that needs or can benefit from these massive parallel executions.

To achieve even better performances, the tau-leaping algorithm was redesigned to 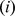 avoid the conditional branches, thus exploiting the underlying SIMD architecture as much as possible, and to 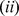 capitalize on the CUDA's memory hierarchy, by pre-calculating some of the needed data structures and by allocating the most used ones into the fastest, yet smallest, memories. Indeed, one the biggest limiting factor for a good occupancy of the CUDA resources is a large use of the shared memory, which can improve the overall performances at the cost of reducing the theoretical occupancy of the SM. Table 3 lists the data type and size of the vectors used by cuTauLeaping. For performances reasons, these vectors are stored into the high-performance memories: vectors containing information that change during the simulation are allocated into the shared memory, while the other information are stored into the constant memory. The dimensions of these vectors are proportional to the number of threads forming a block (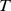), the number of reactions (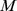), the number of molecular species present in the system (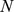) and the number of non-zero entries (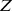) of the corresponding non-flattened stoichiometric matrix.

**Table 3:** tau-leaping data structures residing in CUDA high-performance memories.

*Array name*	*Data type*	*Array size*	*Memory type*
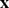	unsigned int (4 bytes)	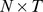	shared memory
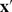	int (4 bytes)	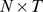	shared memory
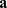	float (4 bytes)	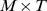	shared memory
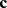	float (4 bytes)	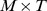	shared memory
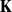	unsigned int (4 bytes)	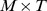	shared memory
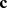	char (1 byte)	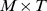	shared memory
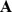	uchar4 (4 bytes)	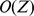	constant memory
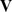	uchar4 (4 bytes)	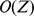	constant memory
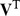	uchar4 (4 bytes)	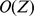	constant memory
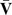	uchar4 (4 bytes)	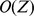	constant memory

Specifically, according to the memory structures described in Table 3, in cuTauLeaping the exact shared memory consumption per SM for a model consisting of 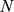 molecular species and 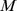 chemical reactions, simulated by 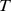 threads per block, is equal to 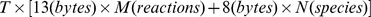. Since the shared memory is a limited resource on the GPU, it follows that the maximum size of a block is proportional to the size of the system, leading to the upper bound
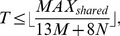
(5)where 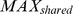 corresponds to the amount of shared memory available on each SM for the specific architecture. According to Equation (5), on a GPU based on the Fermi architecture, the maximum size of a block for the MM, PGN, Schlögl and Ras/cAMP/PKA models corresponds to 692, 381, 646 and 63 threads, respectively. More generally, if we consider a theoretical, very large stochastic model composed by 100 reactions and 100 species, the maximum value for 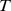 would be 23; multiple blocks can be launched and run on different SMs, if these are available on the GPU, thus allowing a further level of parallelism. For instance, the GeForce GTX 590 that we used in our tests is equipped with 32 SMs and could therefore execute 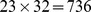 simultaneous threads for this theoretical model. Since the shared memory represents a limiting factor for the parallelism, a subset of the data structures listed in Table 3 might be moved from the shared memory to the slower global memory, thus increasing the 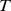 value at the cost of higher latencies in the access to data and of higher computational costs. Being this a relevant aspect of our implementation, we are currently working on the optimization of these data structures, to the purpose of increasing the level of parallelism and further reducing the computational time.

In order to analyze the boost of performances of cuTauLeaping, we compared it with a standard CPU implementation (using the software COPASI [66] as reference), by running several identical simulations of four biological models of increasing size and complexity. Our results showed that tau-leaping running on GPU yields much better results and becomes particularly profitable when a large number of simulations have to be performed. Interestingly, when the number of simulations is limited (ranging from a few units to around one hundred, for the four models we tested), the CPU version may result more efficient than the GPU, being the break-even between the two implementations directly dependent on the complexity of the system and on its emergent dynamics. It is worth noting that, although the computational speedup achieved with cuTauLeaping might be improved by exploiting a faster RNG, such as XORWOW, the results obtained by using the more reliable RNG MRG32K3A still show a relevant reduction of running times with respect to COPASI CPU tau-leaping (see Table 2 in Text S2). Therefore, cuTauLeaping represents an advantageous tool to carry out thorough computational analyses of stochastic biological systems that usually require a huge number of simulations.

**Figure 13:**
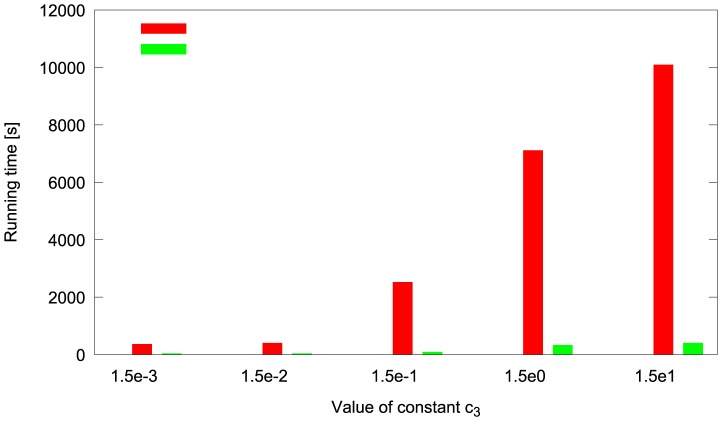
Performance comparison of CPU tau-leaping and cuTauLeaping for a PSA of the Ras/cAMP/PKA model. Running times of cuTauLeaping and COPASI CPU tau-leaping to execute a PSA-1D of the Ras/cAMP/PKA model, where the stochastic constant 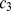 was varied in the interval 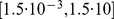 and a total of 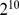 simulations were executed. The plot shows how the computational cost of tau-leaping running on CPU rapidly increases; this behavior can become prohibitive if several independent simulations need to be executed. On the contrary, cuTauLeaping shows a very moderate increase in the running times and outperforms the CPU implementation of tau-leaping.

In addition, we performed different tests on randomly generated synthetic models, suggesting that the inherent complexity of the system and the chosen parameterization are more important than the model size, and they can greatly affect the performances of the simulation algorithm. The variation of the initial parameterization, which is indispensable to carry out a perturbation analysis, usually induces quantitatively and qualitatively distinct dynamical behaviors; even more important is the fact that different parameterizations generally result in different running times, leading to potentially huge and surely unfeasible computational costs, especially when standard CPU executions of stochastic simulation algorithms are performed. As an example to explain this important matter, we compared the running time of cuTauLeaping and of COPASI CPU tau-leaping when varying a single parameter in the Ras/cAMP/PKA model over 5 orders of magnitude (namely, we executed 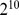 simulations varying the value of the stochastic constant 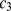 in the sweep interval 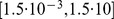). Figure 13 shows that, in this situation, the computational cost of tau-leaping running on CPU rapidly increases; this behavior could become prohibitive if several independent simulations need to be executed. On the contrary, cuTauLeaping shows a very moderate increase in the running times, although following the same growth trend of the CPU counterpart, and outperforms the CPU implementation. An explanation for this behavior is that, being the CPU sequential, a simulation can start as long as the previous one terminated, whilst in the case of GPU the overall running time roughly corresponds to the running time of the slowest simulation. This is particularly relevant in the case of parameterizations leading to high running times on the CPU (e.g., when 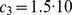 in Figure 13), where the speedup granted by the use of cuTauLeaping is 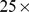, compared to the 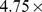 speedup (see Table 1) achieved with the reference parameterization of the Ras/cAMP/PKA model (Table 6 in Text S1).

A *fine-grain* GPU parallelization of tau-leaping was previously proposed in [39], to the aim of accelerating the execution of single runs of tau-leaping. In that work, the computational performances were discussed in relation to very large systems of molecular interactions (with better gains achieved for 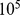 reaction channels), and tested over a *synthetically* generated network consisting of 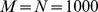 reactions and species. Taking into account the above mentioned issues related to the model parameterization, there is a remarkable aspect that should not be left out when assessing the effective performances of fine-grain implementations of this type. Namely, the synthetic network used in [39] was characterized by a homogeneous initial parameterization (i.e., the values of all stochastic constants were randomly selected with a uniform distribution in 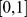), therefore largely limiting the biochemical meaning of the distinct reaction rates that very different molecular interactions – transcription rates, post-translational modification rates, diffusion rates, catalyzed processes, etc. – do actually present in real cellular systems [4], [82]. An arbitrary modification of these values, that are of pivotal importance in the definition and in the analysis of validated models of *real* biological systems, might possibly result in different computational performances of such fine-grain GPU accelerations whenever tested on other well assessed mathematical models of biological systems [83]. This is corroborated by the results that we obtained from the analyses of randomly generated synthetic models, which altogether highlight the impact of the model parameterization on the computational performances: in particular, in cuTauLeaping the highest running times with large standard deviation values were obtained in tests 6 and 8 presented in Table 2, which are characterized by the largest intervals for the choice of stochastic constants and by the highest initial molecular amounts.

As a matter of fact, stochastic modeling and simulation methods are usually assumed to be suitable for relatively small systems (such as signaling pathways), consisting of a few tens of reactions and species, defined according to a bottom-up modeling approach whereby a mechanistic description of the most relevant molecular interactions is provided [18], [84]. The rationale behind this is that a good initial parameterization for models of this type cannot be usually settled by using either literature data or experimental measurements – especially for reaction constants, which are always difficult or even impossible to measure in living systems – and thus a large batch of simulations are generally required not only to analyze the dynamical behavior of the system, but also to corroborate the choice of the initial parameters. Therefore, despite the noticeable boost that a fine-grain GPU-based parallelization of stochastic algorithms can have in terms of single simulations, most of the times the effective requirements for the analysis of real models naturally rely upon *coarse-grain* and massively parallel executions of a large number of simulations, as proposed in this work.

Despite all these possible optimizations, stochastic simulations of complex biological systems still remain a computationally intensive task, especially when some molecular species occur in very low amounts and other in very large amounts – or also when the values of reaction constants span over different orders of magnitude – possibly inducing a slowdown of running time because of stiffness and multi-scale problems. Therefore, future efforts need to be focused on the implementation of GPU-based hybrid simulation algorithms [18], [85]–[87], able to automatically choose the subset of reactions to be simulated with a stochastic algorithm and the subset of reactions for which a deterministic simulation method is more adequate in the analysis of the same biological model.

The cuTauLeaping software is available from the authors upon request.

## Supporting Information

Text S1 **Stochastic biological models.**
(PDF)

Text S2 **Comparison of the computational costs of cuTauLeaping using the random numbers generators XORWOW and MRG32K3A.**
(PDF)
