# Correction: cuTauLeaping: A GPU-Powered Tau-Leaping Stochastic Simulator for Massive Parallel Analyses of Biological Systems

**DOI:** 10.1371/journal.pone.0101858

**Published:** 2014-06-26

**Authors:** The PLOS ONE Staff

The legend within Figure 13 is missing labels. The authors have provided a corrected version here.

**Figure 13:**
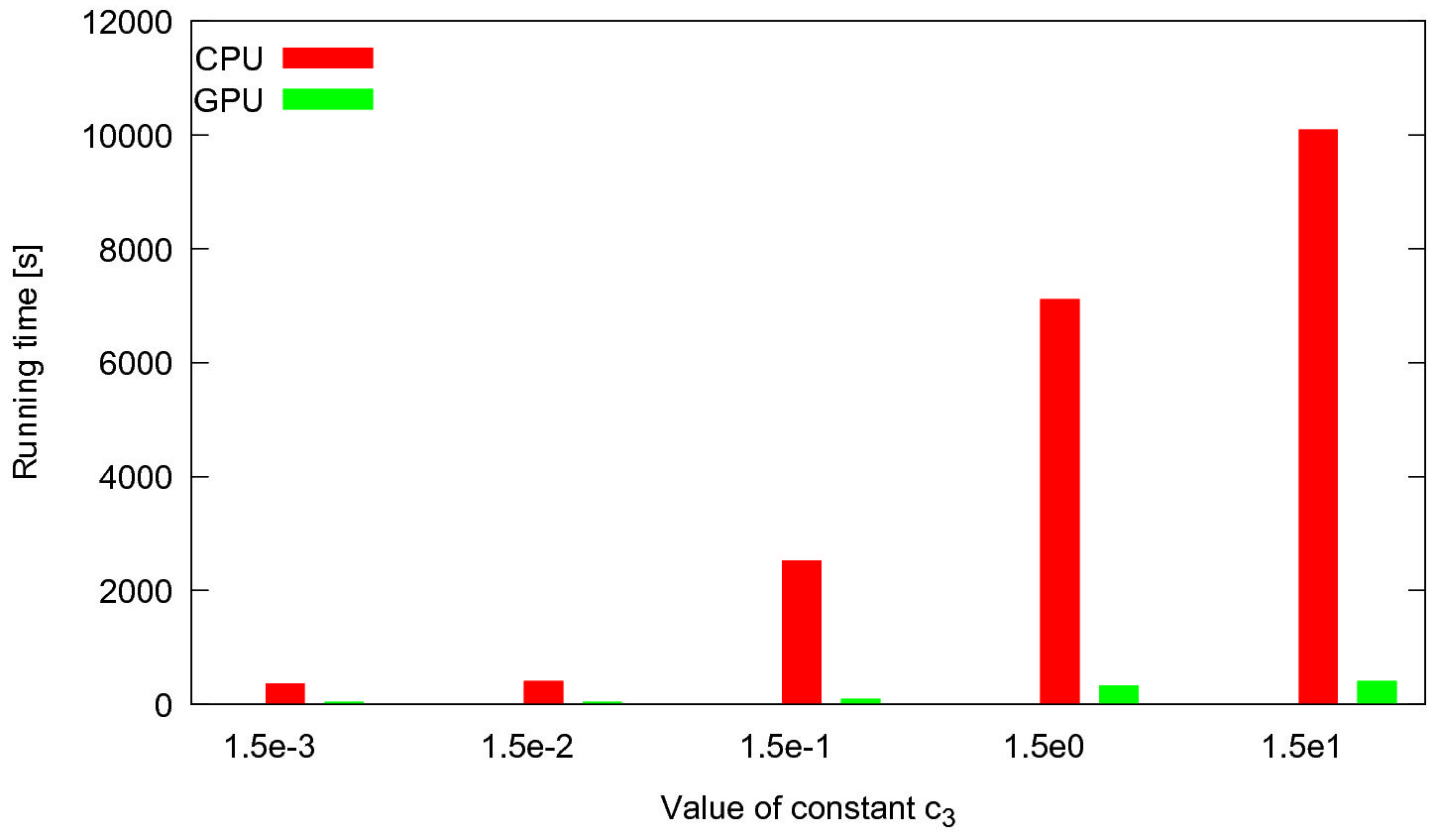
Performance comparison of CPU tau-leaping and cuTauLeaping for a PSA of the Ras/cAMP/PKA model. Running times of cuTauLeaping and COPASI CPU tau-leaping to execute a PSA-1D of the Ras/cAMP/PKA model, where the stochastic constant 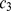 was varied in the interval 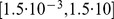 and a total of 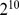 simulations were executed. The plot shows how the computational cost of tau-leaping running on CPU rapidly increases; this behavior can become prohibitive if several independent simulations need to be executed. On the contrary, cuTauLeaping shows a very moderate increase in the running times and outperforms the CPU implementation of tau-leaping.

## References

[1] Nobile MS, Cazzaniga P, Besozzi D, Pescini D, Mauri G (2014) cuTauLeaping: A GPU-Powered Tau-Leaping Stochastic Simulator for Massive Parallel Analyses of Biological Systems. PLoS ONE 9(3): e91963 doi:10.1371/journal.pone.0091963 24663957 10.1371/journal.pone.0091963 PMC3963881

