# Supplementary material for: cuTauLeaping: A GPU-Powered Tau-Leaping Stochastic Simulator for Massive Parallel Analyses of Biological Systems
Source: PLoS One. 2014 Mar 24;9(3):e91963. doi: 10.1371/journal.pone.0091963 (PMC3963881; doi:10.1371/journal.pone.0091963)
Supplement: Text S1 — Stochastic biological models. (PDF) [file pone.0091963.s001.pdf]

Supporting Information - Text S1

Stochastic biological models

1 Michaelis-Menten kinetics

The Michaelis-Menten (MM) model is a simple example of enzyme kinetics consisting in 3 reactions, which describe the binding of an enzyme E to a substrate S to form an intermediate complex ES, that is eventually converted into a final product P [1]. These reactions, together with the values of the associated stochastic constants, are given in Table 1. The initial molecular amounts used in this work, given as number of molecules, are listed in Table 2.

Table 1. Michaelis-Menten model

No.	*Reactants*	*Products*	*Stochastic constant*
r_1	$S + E$	ES	0.0025
r_2	ES	$S + E$	0.1
r_3	ES	$E + P$	5.0

Table 2. Initial molecular amounts in the Michaelis-Menten model

Molecular species	*Initial amount*
S	1000
E	750
ES	0
P	0

2 Prokaryotic auto-regulatory gene network

The prokaryotic gene network (PGN) [2] describes an auto-regulation mechanism of gene expression, whereby a gene (DNA) that codes for a protein (P) is inhibited by binding to a dimer of the protein itself ($DNA{:}P_2$). Gene expression is a good example of stochasticity in biological systems: the transcriptional regulators are present in a few copies, so that the binding and release of the regulators can be expressed in probabilistic terms.

The reactions describing the molecular interactions occurring in PGN, together with the values of the associated stochastic constants, are given in Table 3. The only initial molecular amount used in this work is DNA=200 molecules; all other molecular species are generated in the system as long as reactions are applied.

Table 3. Prokaryotic auto-regulator gene network model

No.	*Reactants*	*Products*	*Stochastic constant*
r_1	$DNA + P_2$	$DNA{:}P_2$	0.1
r_2	$DNA{:}P_2$	$DNA + P_2$	0.7
r_3	DNA	$DNA + mRNA$	0.35
r_4	$mRNA$	λ	0.3
r_5	$2P$	P_2	0.1
r_6	P_2	$2P$	0.9
r_7	$mRNA$	$mRNA + P$	0.2
r_8	P	λ	0.1

Remark: λ denotes the degradation of the reactant

3 The Schlögl system

The Schlögl system [3, 4] is one of the simplest prototypes of chemical systems presenting a bistable dynamical behavior, i.e., the capacity of switching between two different stable steady states in response to some chemical signaling (see, e.g., [5–7] and references therein). The Schlögl model consists of 4 chemical reactions and 3 molecular species, listed in Table 4. The initial molecular amounts used in this work are given in Table 5.

Table 4. The Schlögl model

No.	*Reactants*	*Products*	*Stochastic constant*
r_1	$A+2X$	$3X$	$3 \cdot 10^{-7}$
r_2	$3X$	$A+2X$	$1 \cdot 10^{-4}$
r_3	B	X	$1 \cdot 10^{-3}$
r_4	X	B	3.5

Table 5. Initial molecular amounts in the Schlögl model

Molecular species	*Initial amount*
A	$^*1 \cdot 10^5$
B	$^*2 \cdot 10^5$
X	250

*The amounts of species A, B are kept constant during the execution of simulations. All molecular amounts are expressed as number of molecules.

4 Ras/cAMP/PKA pathway

In the yeast *Saccharomyces cerevisiae*, the Ras/cAMP/PKA pathway plays a major role in the regulation of metabolism, stress resistance and cell cycle progression [8, 9]. This pathway controls more than 90% of all genes that are regulated by glucose through the activation of the protein kinase A (PKA), that is able to phosphorylate a plethora of downstream proteins. PKA is activated by the binding of the second messenger cyclic-AMP (cAMP), which is synthesized by the adenylate cyclase Cyr1. The activity of Cyr1 is controlled by the monomeric GTPases Ras1 and Ras2, which cycle between a GTP-bound active state and a GDP-bound inactive state. In turn, Ras proteins are positively regulated by protein Cdc25, a Ras-GEF (Guanine Nucleotide Exchange Factor) that stimulates the GDP to GTP exchange, and negatively regulated by proteins Ira1 and Ira2, two Ras-GAP (GTPase Activating Proteins) that stimulate the GTPase activity of Ras proteins. The degradation of cAMP is governed by two phosphodiesterases, Pde1 and Pde2. These two enzymes constitute a major negative feedback in this pathway: the low-affinity phosphodiesterase Pde1 is active under the positive regulation of PKA, while the high-affinity phosphodiesterase Pde2 is active in the basal level regulation of cAMP.

The reactions describing the interactions occurring in the Ras/cAMP/PKA pathway, together with the values of the associated stochastic constants, are given in Table 6 (see also [10–12] for further details). In particular:

- reactions $r_1, \ldots, r_{10}$ describe the switch cycle of Ras2 protein between its inactive state (Ras2-GDP) and active state (Ras2-GTP), regulated by the activity of the GEF Cdc25 and of the GAP Ira2;
- reactions r_{11}, r_{12}, r_{13} describe the synthesis of cAMP through the activation of the adenylate cyclase Cyr1, mediated by Ras2-GTP;
- reactions $r_{14}, \ldots, r_{25}$ describe the activation of PKA, mediated by the reversible binding of cAMP to its two regulatory subunits, and the subsequent dissociation of the PKA tetramer, which releases the two catalytic subunits;
- reactions $r_{26}, \ldots, r_{33}$ describe the activity of the two phosphodiesterases Pde1 and Pde2, that carry out the degradation of cAMP. The activation of Pde1 is regulated by the catalytic subunits of PKA, and it represents one of the main negative feedback control exerted by PKA within this pathway;
- reactions r_{34}, r_{35} describe the negative feedback exerted by PKA on Cdc25, whose effect is modeled as a partial inactivation of the GEF activity and a reduction of the active state level of Ras2-GTP.

The initial molecular amounts used in this work are summarized in Table 7.

The SBML version of this model is available at the BioModels database [13] under submission identifier MODEL1309060000.

Table 6. Mechanistic model of the Ras/cAMP/PKA pathway

No.	*Reagents*	*Products*	*Stochastic constant*
r_1	Ras2-GDP + Cdc25	Ras2-GDP-Cdc25	1.0
r_2	Ras2-GDP-Cdc25	Ras2-GDP + Cdc25	1.0
r_3	Ras2-GDP-Cdc25	Ras2-Cdc25 + GDP	1.5
r_4	Ras2-Cdc25 + GDP	Ras2-GDP-Cdc25	1.0
r_5	Ras2-Cdc25 + GTP	Ras2-GTP-Cdc25	1.0
r_6	Ras2-GTP-Cdc25	Ras2-Cdc25 + GTP	1.0
r_7	Ras2-GTP-Cdc25	Ras2-GTP + Cdc25	1.0
r_8	Ras2-GTP + Cdc25	Ras2-GTP-Cdc25	1.0
r_9	Ras2-GTP + Ira2	Ras2-GTP-Ira2	$3.0{\cdot}10^{-2}$
r_{10}	Ras2-GTP-Ira2	Ras2-GDP + Ira2	$7.0{\cdot}10^{-1}$
r_{11}	Ras2-GTP + Cyr1	Ras2-GTP-Cyr1	$1.0{\cdot}10^{-3}$
r_{12}	Ras2-GTP-Cyr1 + ATP	Ras2-GTP-Cyr1 + cAMP	$2.1{\cdot}10^{-6}$
r_{13}	Ras2-GTP-Cyr1 + Ira2	Ras2-GDP + Cyr1 + Ira2	$1.0{\cdot}10^{-3}$
r_{14}	cAMP + PKA	cAMP-PKA	$1.0{\cdot}10^{-5}$
r_{15}	cAMP + cAMP-PKA	(2cAMP)-PKA	$1.0{\cdot}10^{-5}$
r_{16}	cAMP + (2cAMP)-PKA	(3cAMP)-PKA	$1.0{\cdot}10^{-5}$
r_{17}	cAMP + (3cAMP)-PKA	(4cAMP)-PKA	$1.0{\cdot}10^{-5}$
r_{18}	(4cAMP)-PKA	cAMP + (3cAMP)-PKA	$1.0{\cdot}10^{-1}$
r_{19}	(3cAMP)-PKA	cAMP + (2cAMP)-PKA	$1.0{\cdot}10^{-1}$
r_{20}	(2cAMP)-PKA	cAMP + cAMP-PKA	$1.0{\cdot}10^{-1}$
r_{21}	cAMP-PKA	cAMP + PKA	$1.0{\cdot}10^{-1}$
r_{22}	(4cAMP)-PKA	C + C + R-2cAMP + R-2cAMP	1.0
r_{23}	R-2cAMP	R + cAMP + cAMP	1.0
r_{24}	R + C	R-C	$7.5{\cdot}10^{-1}$
r_{25}	R-C + R-C	PKA	1.0
r_{26}	C + Pde1	C + Pde1p	$1.0{\cdot}10^{-6}$
r_{27}	cAMP + Pde1p	cAMP-Pde1p	$1.0{\cdot}10^{-1}$
r_{28}	cAMP-Pde1p	cAMP + Pde1p	$1.0{\cdot}10^{-1}$
r_{29}	cAMP-Pde1p	AMP + Pde1p	7.5
r_{30}	Pde1p + PPA2	Pde1 + PPA2	$1.0{\cdot}10^{-4}$
r_{31}	cAMP + Pde2	cAMP-Pde2	$1.0{\cdot}10^{-4}$
r_{32}	cAMP-Pde2	cAMP + Pde2	1.0
r_{33}	cAMP-Pde2	AMP + Pde2	1.7
r_{34}	C + Cdc25	C + Cdc25p	1.0
r_{35}	Cdc25p + PPA2	Cdc25 + PPA2	$1.0{\cdot}10^{-2}$

Table 7. Initial molecular amounts in the Ras/cAMP/PKA model

Molecular species	*Initial amount*
Cyr1	200
Cdc25	300
Ira2	200
Pde1	1400
PKA	2500
PPA2	4000
Pde2	6500
Ras2-GDP	20000
GDP	$^{*}1.5 \cdot 10^{6}$
GTP	$^{*}5.0 \cdot 10^{6}$
ATP	$^{*}2.4 \cdot 10^{7}$

*The amounts of GDP, GTP and ATP are kept constant during the execution of simulations. All molecular amounts are expressed as number of molecules per cell, derived according to data presented in [14], as described in [10, 12].

References

1. Nelson D, Cox M (2004) Lehninger Principles of Biochemistry. W. H. Freeman Company.

2. Wang Y, Christley S, Mjolsness E, Xie X (2010) Parameter inference for discretely observed stochastic kinetic models using stochastic gradient descent. BMC Syst Biol 4: 99.

3. Vellela M, Qian H (2009) Stochastic dynamics and non-equilibrium thermodynamics of a bistable chemical system: the Schlögl model revisited. J R Soc Interface 6: 925–940.

4. Wilhelm T (2009) The smallest chemical reaction system with bistability. BMC Syst Biol 3: 90.

5. Craciun G, Tang Y, Feinberg M (2006) Understanding bistability in complex enzyme-driven reaction networks. PNAS 103: 8697–8702.

6. Pomerening JR (2008) Uncovering mechanisms of bistability in biological systems. Curr Opin Biotech 19: 381–388.

7. Widder S, Macía J, Solé R (2009) Monomeric bistability and the role of autoloops in gene regulation. PLOS ONE 4: e5399.

8. Santangelo GM (2006) Glucose signaling in *Saccharomyces cerevisiae*. Microbiol Mol Bio Rev 70: 253–282.

9. Zaman S, Lippman SI, Schneper L, Slonim N, Broach JR (2009) Glucose regulates transcription in yeast through a network of signaling pathways. Mol Syst Biol 5: 1–14.

10. Cazzaniga P, Pescini D, Besozzi D, Mauri G, Colombo S, et al. (2008) Modeling and stochastic simulation of the Ras/cAMP/PKA pathway in the yeast *Saccharomyces cerevisiae* evidences a key regulatory function for intracellular guanine nucleotides pools. J Biotechnol 133: 377–385.

11. Pescini D, Cazzaniga P, Besozzi D, Mauri G, Amigoni L, et al. (2012) Simulation of the Ras/cAMP/PKA pathway in budding yeast highlights the establishment of stable oscillatory states. Biotechnol Adv 30: 99–107.

12. Besozzi D, Cazzaniga P, Pescini D, Mauri G, Colombo S, et al. (2012) The role of feedback control mechanisms on the establishment of oscillatory regimes in the Ras/cAMP/PKA pathway in *S. cerevisiae*. EURASIP J Bioinform Syst Biol 10.

13. BioModels Database. URL `http://www.ebi.ac.uk/biomodels-main`.

14. Ghaemmaghami S, Huh W, Bower K, Howson R, Belle A, et al. (2003) Global analysis of protein expression in yeast. Nature 425: 671–672.
