# Supplementary material for: cuTauLeaping: A GPU-Powered Tau-Leaping Stochastic Simulator for Massive Parallel Analyses of Biological Systems
Source: PLoS One. 2014 Mar 24;9(3):e91963. doi: 10.1371/journal.pone.0091963 (PMC3963881; doi:10.1371/journal.pone.0091963)
Supplement: Text S2 — Comparison of the computational costs of cuTauLeaping using the random numbers generators XORWOW and MRG32K3A. (PDF) [file pone.0091963.s002.pdf]

Supporting Information - Text S2

Comparison of the computational costs of cuTauLeaping using the random numbers generators XORWOW and MRG32K3A

The XORWOW algorithm [1] is known to present statistical flaws [2] and therefore should not be exploited to perform Monte Carlo simulations. Nevertheless, it is more performant than the other random numbers generators (RNGs) available in CURAND and, as such, it might be used when a strong reduction of the computational costs is fundamental.

The aim of the tests presented here is to compare the outcomes of stochastic simulations when using XORWOW instead of MRG32K3A [3]. This comparison is carried out on the Schlögl model (see Supporting Information Text S2, Section 3), by executing $2^{10}, 2^{12}, 2^{14}, 2^{16}$ parallel simulations and calculating the frequency distributions of the amount of chemical species X at time $t = 10$ a.u..

In order to verify whether the samples corresponding to XORWOW and MRG32K3A were drawn from the same distribution, we exploited the Kolmogorov-Smirnov (K-S) statistics [4]. When the K-S statistic is small or the p-value is high, the hypothesis that the distributions of the two samples are the same cannot be rejected. Results in Table 1 confirm that, despite XORWOW presents statistical flaws, according to the K-S test the frequency distributions of X obtained with XORWOW and MRG32K3A can be considered completely equivalent. This is also shown in Figure 1, where we can observe that the distributions are perfectly overlapped.

Finally, in Table 2 we report the comparison of the overall running time (expressed in seconds) of different batches of simulations for the four models described in Supporting Information Text S2, executed with a reference CPU implementation of tau-leaping (COPASI [5]) and with cuTauLeaping, using either XORWOW or MRG32K3A. These results show that XORWOW outperforms MRG32K3A in all cases.

Table 1: **Kolmogorov-Smirnov statistics of the frequency distribution of species X in the Schlögl model**

Number of parallel simulations	*K-S test*	*p-value*
2^{10}	0.05	0.9994
2^{12}	0.04	0.9999
2^{14}	0.04	0.9999
2^{16}	0.05	0.9994

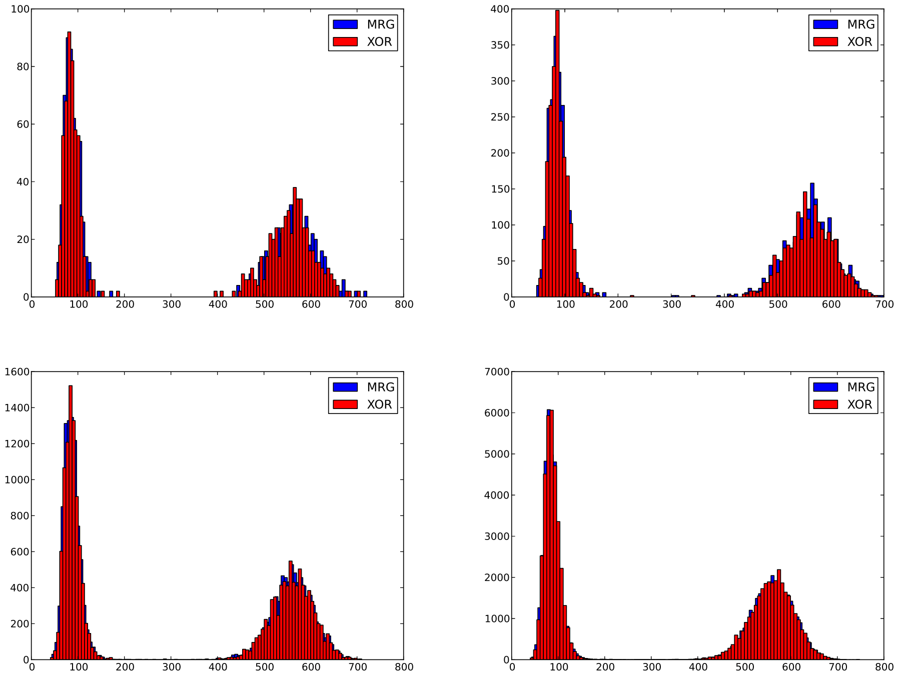

Figure 1: Comparison of the frequency distribution of the amount of chemical species X of the Schlögl model at time $t = 10$ a.u., using MRG32K3A (blue) and XORWOW (red). Top-left: 2^{10} simulations; top-right: 2^{12} simulations; bottom-left: 2^{14} simulations; bottom-right: 2^{16} simulations.

Table 2: **Computational performance of cuTauLeaping using different random numbers generators**

Model	*Simulations*	*CPU time*	*GPU time* (*XORWOW*)	*GPU time* (*MRG32K3A*)	*Speedup* (*XORWOW*)	*Speedup* (*MRG32K3A*)
Michaelis-Menten model	2^6	0.047	0.035	0.038	1.34 ×	1.23 ×
	2^8	0.219	0.038	0.046	5.75 ×	4.75 ×
	2^{10}	0.905	0.044	0.047	20.56 ×	19.25 ×
	2^{12}	4.087	0.049	0.051	83.41 ×	80.14 ×
	2^{14}	19.001	0.064	0.073	296.89 ×	260.29 ×
	2^{16}	76.691	0.141	0.172	543.91 ×	445.87 ×
	2^{18}	309.241	0.406	0.530	761.68 ×	583.47 ×
Prokaryotic gene network	2^6	0.468	0.117	0.120	4.00 ×	3.90 ×
	2^8	1.997	0.117	0.121	17.07 ×	16.50 ×
	2^{10}	8.112	0.123	0.124	65.95 ×	65.42 ×
	2^{12}	32.807	0.132	0.128	248.54 ×	256.30 ×
	2^{14}	130.885	0.172	0.175	760.96 ×	747.91 ×
	2^{16}	526.535	0.55	0.591	957.34 ×	890.92 ×
	2^{18}	2095.48	2.05	2.18	1022.2 ×	961.23 ×
Schlögl model	2^6	0.202	0.725	0.723	0.27 ×	0.28 ×
	2^8	0.811	0.790	0.875	1.02 ×	0.92 ×
	2^{10}	3.603	0.896	0.979	4.02 ×	3.68 ×
	2^{12}	13.993	1.132	1.156	12.36 ×	12.10 ×
	2^{14}	56.254	1.265	1.578	44.46 ×	35.64 ×
	2^{16}	224.454	2.113	3.534	106.22 ×	63.51 ×
	2^{18}	905.664	5.644	10.163	160.46 ×	89.11 ×
Ras/cAMP/PKA pathway	2^6	118.873	303.5	320.1	0.39 ×	0.37 ×
	2^8	445.632	299.56	322.4	1.49 ×	1.38 ×
	2^{10}	1769.58	327.4	372.4	5.40 ×	4.75 ×
	2^{12}	8828.05	515.7	551.4	17.12 ×	16.01 ×
	2^{14}	35027.9	1376.5	1530.1	25.45 ×	22.89 ×
	2^{16}	133733	4898	5482	27.30 ×	24.39 ×
	2^{18}	534932*	19004	21470	28.15 ×	24.92 ×

*Estimated value

References

[1] Marsaglia G (2003) Xorshift RNGs. J Stat Softw 8: 1–6.

[2] Hill DRC, Mazel C, Passerat-Palmbach J, Traore MK (2013) Distribution of random streams for simulation practitioners. Concurr Comp-Pract E 25: 1427–1442.

[3] L'Ecuyer P, Simard R, Chen EJ, Kelton WD (2002) An object-oriented random-number package with many long streams and substreams. Oper Res 50: 1073–1075.

[4] Gentle J (2003) Random Number Generation and Monte Carlo Methods. Statistics and Computing. Springer.

[5] Hoops S, Sahle S, Gauges R, Lee C, Pahle J, et al. (2006) COPASI - a COmplex PAthway SImulator. Bioinformatics 22: 3067-3074.
